# A randomized, double-blind, placebo-controlled phase III clinical trial to evaluate the efficacy and safety of SARS-CoV-2 vaccine (inactivated, Vero cell): a structured summary of a study protocol for a randomised controlled trial

**DOI:** 10.1186/s13063-021-05180-1

**Published:** 2021-04-13

**Authors:** Murat Akova, Serhat Unal

**Affiliations:** Department of Infectious Diseases and Clinical Microbiology, Hacettepe University School of Medicine, Hacettepe Mh., 06230 Ankara, Turkey

**Keywords:** COVID-19, randomised placebo controlled trial, double blind, protocol, inactivated vaccines, phase III clinical trial, COVID-19, vaccine, Turkey

## Abstract

**Objectives:**

The primary objective is to evaluate the efficacy of an inactivated and aluminium hydroxide adsorbed SARS-CoV-2 vaccine (Sinovac, China) in voluntary participants after 14 days of the second dose against RT-PCR confirmed symptomatic COVID-19 cases. The secondary objectives include evaluating the efficacy after at least one dose of the vaccine against RT-PCR confirmed symptomatic COVID-19 cases; the efficacy of two doses of the vaccine on the rates of hospitalization and death; the safety of the vaccine including adverse reactions up to one year after the 2^nd^ dose of vaccination; and the immunogenicity of the vaccine and its duration up to 120 days.

**Trial Design:**

This is a phase III, randomized, double-blind, placebo-controlled case driven clinical trial to assess the efficacy and safety of the vaccine. The study is planned to be carried out within two separate cohorts in voluntary participants aged between 18-59 years old. The first cohort includes healthcare professionals actively working in healthcare units, who are assumed to have higher risk of acquiring COVID-19, and the second cohort includes other immunocompetent subjects in the same age group, who are at a regular risk for COVID-19 disease. In Cohort 1, healthcare professionals will be randomized to receive two intramuscular doses of investigational product or the placebo in a 1:1 ratio and they will be monitored for 12 months by active surveillance of COVID-19. In Cohort 2, immunocompetent subjects will be randomized to receive vaccine or the placebo in a 2:1 ratio.

**Participants:**

Healthcare professionals of both genders, including medical doctors, nurses, cleaners, hospital technicians, and administrative personnel who work in any department of a healthcare unit and immunocompetent individuals of both genders are included. Pregnant (confirmed by positive beta-hCG test) and breastfeeding women as well as those intending to become pregnant within three months after vaccination are excluded. Other exclusion criteria include history of COVID-19 test positivity (PCR or immunoglobulin test results), any form of immunosuppressive therapy including corticosteroids within 6 months, history of bleeding disorders, asplenia, and administration of any form of immunoglobulins or blood products within 3 months.

Exclusion criteria for the second dose include any serious adverse events related with the vaccine, anaphylaxis or hypersensitivity after vaccination, or any confirmed or suspected autoimmune or immunosuppressive disease (including HIV infection).

Participants are only included after signing the voluntary informed consent form, ensuring cooperation in visits, undergoing screening for evaluation, and conforming to all the inclusion and exclusion criteria. All clinical sites are located in Turkey.

**Intervention and comparator:**

The vaccine was manufactured by Sinovac Research & Development Co., Ltd. It is a preparation made from a novel coronavirus (strain CZ02) grown in the kidney cell cultures (Vero Cell) of the African green monkey and contains inactivated SARS-CoV-2 virus, aluminium hydroxide, disodium hydrogen phosphate, sodium dihydrogen phosphate, and sodium chloride. A dose of 0.5 mL contains 600 SU of SARS-CoV-2 virus antigen. The placebo contains aluminium hydroxide, disodium hydrogen phosphate, sodium dihydrogen phosphate, and sodium chloride (0.5mL/dose).

Scheduled visits and additional unscheduled weekly visits will be performed for the first 13 weeks and neutralizing antibody test, IgG test, T-Cell activation test, pregnancy test, and RT-PCR tests along with total antibody test will be performed. Adverse events and serious adverse events during the follow-up will be recorded on diary cards. Diary cards will collect information on the timing and severity of COVID-19 symptoms and solicited adverse events recorded by the subjects during one-year follow-up period. All serious adverse events will be managed and necessary treatment will be ensured according to the local regulations. All serious adverse events following vaccination will be reported to the ethics committee, the Ministry of Health, and the study sponsor within 24 hours of detection.

**Main Outcomes:**

The primary efficacy endpoint is the incidence of symptomatic cases of COVID-19 disease confirmed by RT-PCR two weeks after the second dose of vaccination. Secondary efficacy endpoints are the incidence of hospitalization/mortality rates among one or two dose regimens, duration of immunogenicity rates up to 120 days, the seroconversion rate, the seropositivity rate, neutralizing antibody titer, and IgG levels 14 days after each dose of vaccination. The primary safety endpoint is the severity and frequency of local and systemic adverse reactions during the period of one week after vaccination. The study would be terminated if more than 15% of the subjects have grade ≥3 adverse events related to vaccination including local reactions.

**Randomisation:**

Eligible subjects will be randomized at their Study Day 0 to two study groups using an Interactive Web Response System (IWRS; developed by Omega CRO, Ankara, Turkey) in both risk groups. The IWRS system customizes the randomization algorithm. After enrolment in the study, each participant will be randomly assigned to either of the two treatment arms at a ratio of 1:1 in the high-risk group and at a ratio of 2:1 in the normal risk group. Each enrolled participant will be assigned to a code and will receive the treatment labelled with the code.

**Blinding (masking):**

The trial is a double-blind study to avoid introducing bias. The blinding may be broken by the investigator in the event of a medical emergency in which knowledge of the identity of the study vaccine is critical for management of the subject’s immediate treatment. The Data and Safety Monitoring Board is to be contacted in case of breaking the blinding for a study object. The blood samples will be taken from both placebo and vaccinated groups, in order not to break the blinding.

**Numbers to be randomised (sample size):**

The study is planned to be carried out with two separate cohorts. The Cohort 1 includes healthcare professionals working in healthcare units and the Cohort 2 consists of immunocompetent subjects having normal risk for COVID-19 disease. The Cohort 2 will be initiated after the evaluation of the interim safety report of the Cohort 1 by the Data and Safety Monitoring Board. Both cohorts will be followed-up via RT-PCR to confirm symptomatic COVID-19 cases. If the clinical efficacy of the vaccine is shown in the Cohort 1 or 2, the subjects randomized into the placebo arm will also be vaccinated. In the Cohort 1, 588 subjects should be included in both arms with the assumption that the risk of infection with COVID-19 will be 5% for the placebo arm and 2% for the vaccine arm in the high-risk group. Considering 10% of drop-out rate and 5% of seropositivity or PCR positivity at baseline, 680 subjects should be screened at both arms of the Cohort 1. Group sample sizes of 7545 SARS-CoV-2 vaccine and 3773 placebo suits at a two-sided 95% confidence interval for the difference in population proportions with a width equal to 1.0%, when the estimated incidence rate for vaccinated group is 1.0% and the estimated incidence rate for placebo group is 2.0%. Drop-out rate is assumed to be 10% and seropositivity or PCR positivity at baseline is assumed to be 5%; accordingly, 13000 participants are needed to be enrolled totally in both cohorts. The remaining 11640 subjects will be screened in the Cohort 2 and eligible subjects will be randomized at a ratio of 2:1.

**Trial Status:**

Protocol version 6.0 – 15 October 2020. Recruitment started on 15.09.2020 and is expected to end on February 2022.

**Trial Registration:**

ClinicalTrials.gov, NCT04582344. Registered 8 October 2020

**Full Protocol:**

The full protocol of the trial is attached as an additional file, accessible from the Trials website (Additional file 1). In the interest in expediting dissemination of this material, the familiar formatting has been eliminated; this Letter serves as a summary of the key elements of the full protocol.

**Supplementary Information:**

The online version contains supplementary material available at 10.1186/s13063-021-05180-1.

## Supplementary Information

**Additional file 1.** Full Study Protocol.

## Data Availability

Not applicable.

