# Supplementary material for: A randomized, double-blind, placebo-controlled phase III clinical trial to evaluate the efficacy and safety of SARS-CoV-2 vaccine (inactivated, Vero cell): a structured summary of a study protocol for a randomised controlled trial
Source: Trials. 2021 Apr 13;22:276. doi: 10.1186/s13063-021-05180-1 (PMC8042350; doi:10.1186/s13063-021-05180-1)
Supplement: Supplementary file 1 — Additional file 1. Full Study Protocol. [file 13063_2021_5180_MOESM1_ESM.docx]

| **Randomized, Double-Blind, Placebo-Controlled Phase III Clinical Trial For Evaluation of Efficacy and Safety of SARS-CoV-2 Vaccine (Vero cell), Inactivated** |
| --- |

| **Protocol** | |
| --- | --- |
| **Protocol Version No:** | 6 |
| **Protocol Date:** | 15.10.2020 |
| **Protocol Code** | 9026-ASI |
| **Country coordinator** | Prof. Dr. Serhat Ünal |
| **Coordinator:** | Prof. Dr. Murat Akova |
| **Site of Coordinator:** | Hacettepe University, Faculty of Medicine, Infectious Diseases and Clinical Microbiology Department |

Contents

[1. Background and PrIncIple 3](#_Toc53749982)

[1.1. Abstract 3](#_Toc53749983)

[1.2. Virology 3](#_Toc53749984)

[1.3. Clinical Manifestations 4](#_Toc53749985)

[1.4. Epidemiologic feature 4](#_Toc53749986)

[1.5. R&D of Vaccines 6](#_Toc53749987)

[1.5.1. Preclinical Study and Laboratory Evaluation of Inactivated SAR-CoV-2 Vaccines 6](#_Toc53749988)

[1.5.2. Immunogenicity study 12](#_Toc53749989)

[1.5.3. Study of protection against virus attack 15](#_Toc53749990)

[1.5.4. Study of Cross Neutralization 20](#_Toc53749991)

[1.6. Clinical trial of SARS-CoV-2 vaccine in healthy adults 20](#_Toc53749992)

[1.7. Product features 21](#_Toc53749993)

[1.7.1. Preparation process and Formula of vaccine 21](#_Toc53749994)

[1.7.2. Placebo vaccine 22](#_Toc53749995)

[1.7.3. Storage and Transportation 22](#_Toc53749996)

[1.7.4. Dosage and administration: 22](#_Toc53749997)

[1.7.5. Information of Investigational vaccine 22](#_Toc53749998)

[1.7.6. Inactivation process 23](#_Toc53749999)

[2. ObjectIves 23](#_Toc53750000)

[3. Study desIgn/type 24](#_Toc53750001)

[3.1.1. Randomization and Unblinding 24](#_Toc53750002)

[3.1.2. Sample Size 25](#_Toc53750003)

[3.1.3. Eligibility criteria 26](#_Toc53750004)

[3.1.4. Withdrawal of Participation 28](#_Toc53750005)

[3.1.5. Termination of the Study 29](#_Toc53750006)

[3.1.6. Study Method 29](#_Toc53750007)

[3.1.7. Follow-up Contacts 32](#_Toc53750008)

[3.1.8. Visits for placebo arm 33](#_Toc53750009)

[3.1.9. Procedures in The Event of Fever and Suspicion of COVID-19 33](#_Toc53750010)

[3.1.10. COVID-19 CASE DEFINITIONS 34](#_Toc53750011)

[3.1.11. Clinical progression scale for SARS-CoV-2 infection 36](#_Toc53750012)

[3.1.12. Procedure in The Event of Loss of Visit or Contact 36](#_Toc53750013)

[4. SurveIllance for Adverse events 41](#_Toc53750014)

[5. Safety Assessment 45](#_Toc53750015)

[6. Data Safety MonItorIng Plan (DSMP) 51](#_Toc53750016)

[7. EthIcal Assurance for ProtectIon of Human rIghts 56](#_Toc53750017)

# Background and PrIncIple

## Abstract

Since December 8, 2019, Hubei Province has reported several cases of unexplained pneumonia, most of whom work or live in the South China seafood market where live animal sales exist. The early stage of pneumonia presents severe symptoms of acute respiratory infection, and some patients develop rapidly into acute respiratory distress syndrome (ARDS). The pneumonia was confirmed to be human to human transmission, and the epidemic escalated rapidly in early January. There were cases in China's provinces, Japan, Singapore, the United States and more than 20 countries. A novel coronavirus was detected in the throat swab samples of patients in January 7, 2020 by the China Center for Disease Control and Prevention (CDC). The novel coronavirus pneumonia epidemic was declared a public health emergency in January 31, 2020 by WHO. In March 11, 2020, WHO declared the epidemic entered the international pandemic stage.

The novel coronavirus gene sequences are most closely related to the two SARS like coronavirus (bat-SL-CoVZC45 and bat-SL-CoVZXC21) ^[6]^ derived from bat. The International Committee on Taxonomy of viruses novel coronavirus was officially classified in February 12, 2020 by the International Committee on Taxonomy of Viruses (ICTV), known as Severe Acute Respiratory Syndrome Coronavirus 2 (SARS-CoV-2), and the World Health Organization (WHO) announced on the same day that the official name of the disease caused by the virus is COVID-19.

## Virology

Coronavirus (COV) is an important pathogen of human and vertebrate. It can infect respiratory tract, gastrointestinal tract, liver and central nervous system of human, livestock, birds, bats, mice and many other wild animals. Since the outbreak of severe acute respiratory syndrome (SARS) in 2003 and the outbreak of Middle East respiratory syndrome (MERS) in 2012, the possibility of CoVs spreading from animals to humans has been proved. CoVs belong to the coronavirinae family of Nidovirales coronavirus family, which includes four genera: α-coronavirus, β- oronavirus, γ-coronavirus and δ-coronavirus ^[7]^.

The novel coronavirus belongs to β- oronavirus, with envelop and round or oval shape, and is usually polymorphic, with a diameter of 60-140nm, and a positive strand RNA virus. The genetic characteristics of SARSr-COV and MERSr-COV are significantly different. At present, the homology with bat-SL-COVZC45 is more than 85%. SARS-CoV-2 can be found in respiratory epithelial cells in 96 hours in vitro, while it takes about 6 days in Vero E6 and Hun-7cell lines.

So far, the whole genome sequences of virus samples are almost the same, suggesting that there is no obvious variation of virus. Close monitoring of novel coronavirus also showed that no significant variation was found in the virus isolated from the environment or in the early stage of the virus isolated from the human body to the recently isolated virus. However, mutation or recombination may occur in the future, and toxicosis may increase or decrease in the mutation process.

The understanding of the physical and chemical characteristics of coronavirus mostly comes from the study of SARS-COV and MERS-COV. The virus is sensitive to UV and heat. 56 ℃ for 30 minutes, ether, 75% ethanol, chlorine containing disinfectant, peracetic acid and chloroform can effectively inactivate the virus, while chlorhexidine cannot effectively inactivate the virus ^[9]^.

## Clinical Manifestations

Based on the current epidemiological survey, the incubation period is 1-14 days, mostly 3-7 days, with fever, fatigue and dry cough as the main manifestations. A small number of patients with nasal obstruction, runny nose, sore throat, myalgia and diarrhea and other symptoms. Severe patients often have dyspnea and/or hypoxemia one week after the onset of the disease. The severe patients rapidly progress to acute respiratory distress syndrome, septic shock, metabolic acidosis and coagulation dysfunction which are difficult to correct. It should be noted that the course of severe and critical patients may be moderate to low fever, or even no obvious fever.

The symptoms of some children and newborns are not typical, such as vomiting, diarrhea and other gastrointestinal symptoms, or only mental weakness, shortness of breath. Mild patients only showed low fever and fatigue, no pneumonia.

Most patients have a good prognosis, and a few are in critical condition. The prognosis of the elderly and those with chronic basic diseases is poor. The clinical manifestations between pregnant women and non-pregnant were similar.

## Epidemiologic feature

**Transmission route and Susceptible population**

The main source of novel coronavirus pneumonia is new crown virus infection, asymptomatic infection may also become a source of infection. Respiratory droplets and contact transmission are the main routes of transmission. The virus is transmitted through the droplets produced by patients' coughing, sneezing and talking. The susceptible people are generally susceptible to infection after inhalation. In a relatively closed environment exposed to high concentration aerosol for a long time, there is the possibility of aerosol propagation.

The route of fecal oral transmission remains to be determined. Recently, novel coronavirus was detected in feces of patients diagnosed in Wuhan, Shenzhen and even the United States. It indicated that the virus could be duplicated and existed in the digestive tract, suggesting that there was a possible [10] of fecal oral transmission. However, it was not clear that the infection and transmission of food contaminated by viral contamination could not be determined. It is also believed that the virus in the feces may be redistributed through the formation of aerosols by droplets containing the virus, which needs further investigation and research.

The novel coronavirus pneumonia is reported to be diagnosed by the mother. The new positive coronavirus may cause infection in the newborn by 30h^[11]^.

**Epidemic situation of COVID-19 in China**

As of 10:00 (CEST) May 18, 2020, there have been 84,494 confirmed cases and 4,645 deaths in China [13]. China-WHO Novel Coronavirus Pneumonia (COVID-19) Joint Investigation Report [14] pointed out that of 55,924 confirmed patients, the median age is 51 years (2 days to 100 years), and the interquartile spacing is 39 to 63 years old. 77.8% of patients are aged between 30 and 69 years old. Among them, 51.1% are male, 77% are from Hubei Province, 21.6% are farmers or manual workers. 2114 confirmed case died, with a crude mortality rate of 3.8%, lower than that of SARS in 2003. The crude mortality rate varies by region and transmission intensity (5.8% in Wuhan and 0.7% in the rest of China). The elder of the age, and the higher of the mortality rate. The mortality rate of patients over 80 years old was the highest (21.9%), and that of men was higher than that of women (4.7% and 2.8%, respectively). The crude mortality rate of retirees was the highest (8.9%) among all professions; the crude mortality rate of patients without complications was 1.4%; for those with complications was significantly higher, 13.2% in cardiovascular patients, 9.2% in diabetes, 8.4% in hypertension, 8.0% in chronic respiratory patients and 7.6% in cancer. The clinical manifestations of children are mild. Only a few cases under the age of 19 developed into severe or critical diseases, accounting for 2.5% and 0.2% respectively.

In China, person-to-person transmission of novel coronavirus pneumonia occurs mainly in families according to the cluster case investigation and some family transmission studies in several provinces. A total of 1836 reported cases from Guangdong Province and Sichuan Province, of them1,308 patients were reported in 344 clusters, 78% - 85% of which occurred in family members. The research for family internal transmission is in progress, but the preliminary results in Guangdong Province estimate that the second attack rate of family members is about 3%-10%. As the epidemic continues, although familial cluster infection dominates, community cluster infection also increases within hospital [14].

**Global Epidemic situation of COVID-19**

As of 10:00 (CEST) on May 18th 2020, there have been 4,618,821 confirmed cases and 311,847 deaths were reported globally. Countries with high incidence rate are America (1,432,265 confirmed cases), Russia (290,678 confirmed cases), UK (243,699 confirmed cases), Spain (231,350 confirmed cases), Italy (225,435 confirmed cases), Germany (174,697 confirmed cases), Turkey (149,435 confirmed cases), France (140,036 confirmed cases), and so on. The outbreak has influenced 212 countries all around the world, and has caused global COVID-19 pandemic

## R&D of Vaccines

So far, no specific therapies or vaccines have been approved for COVID-19. According to WHO, there are 119 candidate vaccines in R&D stage, 110 candidates are in the preclinical stage, 9 candidates are under clinical trials, from which 1 candidate in phase I clinical trial, 2 candidates in phase II, and 6 candidates in phase I/II clinical trial. According to the technical classification, 48 of the candidate vaccines are recombinant protein vaccines (mainly sub unit vaccines and virus-like particles), 30 are nucleic acid vaccines (including 20 mRNA vaccines and 10 DNA vaccines), and 27 are viral vector vaccines (using 9 vectors, including adenovirus, influenza virus, poxvirus, measles virus, vesicular stomatitis virus, yellow fever virus, rabies virus, Newcastle disease virus and avian paramyxovirus), 8 are inactivated vaccines (under research in China, Japan, the United States and Kazakhstan), 2 are live attenuated vaccines (under research in India), and 4 are other vaccines (therapeutic vaccines).

### Preclinical Study and Laboratory Evaluation of Inactivated SAR-CoV-2 Vaccines

**Safety study**

The Single dose toxicity test, Systemic active anaphylaxis test in guinea pigs, Repeated dose toxicity test on rat, Repeated dose toxicity test on Macaca monkey and Reproductive development toxicity test were carried out for the inactivated SARS-CoV-2 vaccine. The results are as follows:

***Single dose toxicity test on rat***

Objective: To evaluate the acute toxicity of the inactivated SARS-CoV-2 (Vero Cell) vaccine in Sprague-Dawley (SD) rats after a single intramuscular injection, and to provide animal experimental data for the follow-up study.

Test design: according to the animal weight measured before administration, 20 animals with similar weight and qualified quarantine were selected for the test, half male and half female; the animals were randomly divided into two groups according to the gender section, namely the test article group and the negative control group. The rats in the test group were given 0.5mL/1200SU/animal high-dose vaccine by intramuscular injection, and the rats in the negative control group were given 0.5mL/saline/animal by intramuscular injection. The rats in the test group were observed for 14 days after the administration, and the gross anatomy was further observed.

**Results:** No death or near-death rats was observed in two groups, and also no clinical abnormal reaction was observed. The body weight in each group showed a normal increasing trend. Compared with the rats in control group of same gender in the same period, there was no statistical difference in the body weight, and there was no significant effect on the food intake due to the vaccination. Results from anatomical observation shows no abnormality in the main organs and tissues was observed in each group.

**Conclusion:** No abnormal changes related to vaccination were observed during the study when given high dosage vaccine on SD rats which intended for clinical use, and the maximum tolerated dose (MTD) of SD rats was greater than or equal to 1200SU/dose.

***Systemic active anaphylaxis test in guinea pigs***

Objective: To observe the rapid systemic anaphylaxis after guinea pigs were sensitized by intramuscular injection of the inactivated SARS-CoV-2 vaccine (once every other day, three times in total) and challenged by intravenous injection of D19 and/or D26, so as to provide animal experimental data for the clinical study of the investigational vaccine.

Test design: according to the animal weight measured before administration, 36 Hartley guinea pigs with similar body weight were randomly divided into four groups: low dose group, high dose group, negative control group and positive control group.

Muscle sensitization was conducted on the 1^st^, 3^rd^ and 5^th^ day with 0.5mL/1200SU/dose of test sample, physiologic saline and human hemoglobin, respectively (14 days after the last sensitization) and on the 26^th^ day, the first three animals in each group were injected intravenously, and the stimulation dose in each group was twice of the sensitization dose. Clinical observation was carried out after administration. The test design is shown in the following table:

**Table** **1 Test design of Systemic active anaphylaxis test in guinea pigs**

| Group | Test sample/  Control | Qty. of Animals | Sensitization (i.m)  D1, D3, D5 | | Stimulation (i.v)  D19, D26 | |
| --- | --- | --- | --- | --- | --- | --- |
|  |  |  | Administration dosage | Dosage capacity (mL/animal) | Administration dosage | Dosage capacity (mL/animal) |
| 1 | Negative control | 4 | 0 | 0.5 | 0 | 1 |
| 2 | Positive control | 4 | 20 mg/animal | 0.5 | 40 mg/ animal | 1 |
| 3 | Low dose of test sample | 4 | 0.1dose/ animal | 0.05 | 0.2 dose / animal | 0.1 |
| 4 | High dose of test sample | 4 | 1dose/ animal | 0.5 | 2 dose / animal | 1 |

Results: there was not abnormal reaction in general clinical observation. The weight of each group was measured before grouping, before the last sensitization and before the administration on the day of stimulation, and the weight growth of each group was normal. The anaphylaxis was negative in the low dose group, high dose group and negative control group, and positive in the positive control group on the 19^th^ and 26^th^ day.

Conclusion: No allergic reaction was observed during the study when given high dosage vaccine to the guinea pigs

***Repeated dose toxicity test on rat***

Objective: To evaluate the possible toxic reactions and target organs of SD rats after repeated intramuscular injection of the inactivated SARS-CoV-2 vaccine for 4 weeks, and the recovery of toxic reactions after 4 weeks of discontinuation, so as to provide animal experimental data for the clinical study of test samples.

**Design of the three-doses schedule**: according to the animal weight measured before grouping, 150 animals with qualified quarantine and similar body weight were selected and randomly divided into 7 groups according to the gender section, which were used in the main test group (1-4 groups, low-dose group of test sample, high-dose group of test sample, negative control group and adjuvant control group) and satellite group (5-7 groups, low-dose group of test sample, high-dose group of test sample and negative control group). There were 15 animals of each sex in the main experimental group, 15 animals of each sex in the satellite group and 5 animals of each sex in the satellite group. The low-dose group, high-dose group, negative control group and adjuvant control group were treated with 0.5mL/300SU/dose, 0.5mL/1200SU/dose of test samples, 0.5mL/dose of normal saline, 0.5mL/dose of adjuvant respectively. The safety of inactivated SARS-CoV-2 vaccine was observed by intramuscular injection on the 1^st^, 8^th^ and 15^th^ day until 4 weeks after the last administration. The test indexes include: clinical observation of allergic reaction, injection local reaction, body weight/body temperature/food/ophthalmic examination, clinical pathological indexes (blood cell count, coagulation function, blood biochemistry, urinalysis), immunological indexes (T-lymphocyte subsets, cytokines, antibodies) and pathological examination (gross anatomy observation, histopathology examination).

**Design of the four-doses schedule:** A total of 80 SD rats aged 5~6 weeks with equal gender and weight, were selected and randomized into 2 groups, i.e. negative control group and vaccine group. There were 40 animals in each group, with 30 in the main group and 10 in the satellite group. All the animals were administered intramuscularly on the week 0,1,2,3 with 0.5ml/1200SU/dose. The recovery period is 2 weeks, and the test indicators were same as that of the study of the 3 dose schedule.

**Results of the three-doses schedule:** No death or near-death rats was observed in 7 groups, and also no clinical abnormal reaction was observed. The body weight in each group showed a normal increasing trend during the study period. No abnormalities were observed in body temperature and eye examinations. No abnormalities in blood coagulation indicators, blood biochemical indicators, urine test indicators, T lymphocyte subsets and cytokines related to drug administration were observed. Compared with the rats in negative control group of same gender in the same period, on Day 4, basophils elevated among female rats in the low dosage group. And 3 days post last dose, neutrophils elevated among male rats and eosinophils elevated among female rats in the low dosage group, and neutrophils elevated among male rats in the high dosage group. Considering the mode and mechanism of the reaction, it’s suggested that those changes may be related to the immune responses and/or local irritation induced by the test sample. In addition, compared with the rats in negative control group of same gender in the same period, 3 days post last dose, lymphocytes elevated among male rats in the low dosage group, while decreased in adjuvant groups. At the end of the convalescent period (Day 29), reticulocyte decreased among female rats in adjuvant group, monocytes elevated among female rats in the low dosage group. Since all those changes were not significant and only observed in a single gender, which indicated there was no correlation with the dose, it is not considered to have toxicological significance. As the increasing of the doses and time, the number of the animals producing antibodies and the titer of the antibodies increased. One week after the 2 doses and two weeks after the 3 doses, in the low dosage and high dosage group, the seroconversion rates of neutralizing antibody and IgG antibody were all 100%, the geometric mean titer (GMT) of the neutralizing antibody was 1:3162 and 1:5103 respectively, and GMT of the IgG antibody was 1:147033 and 1:294067 respectively.

Results from pathological examination showed that no regular changes of pathological significance in organ weight and organ coefficient of animals in each group were observed, no significant changes in gross observation was found. Microscopic observation was conducted 3 days post administration (Day 18), local granulomatous inflammation was observed in 17/20, 13/20 and 10/20 of rats in adjuvant group, low dosage group and high dosage group, respectively, with pathological changes ranging from mild to moderate. The change was considered to be local reaction induced by aluminum adjuvant, which belongs to the expected reaction induced by intramuscular injection of aluminum-containing vaccine. At the end of the 2-week convalescent period (Day 29), local granulomatous inflammation was observed in 7/10, 6/10 and 6/10 of rats in adjuvant group, low dosage group and high dosage group, respectively, indicating that the local irritation reaction of administration had not yet recovered.

Results of the four-doses schedule: No death or near-death rats was observed in 2 groups, and also no clinical abnormal reaction was observed. No abnormalities were observed in body temperature and eye examinations. At the end of the last administration and the end of the recovery period, there were no vaccination related abnormalities were observed in coagulation function indicators, blood biochemical indicators, urine test indicators, T lymphocyte subsets associated indicators, nor cytokines (IL-2, IL-10, and TNF-α) indicators.

At the end of the last dose vaccination, the serum IL-2 and TNF-α of the male and female animals in negative control group and vaccine group were below the lowest detection limit. Only one animal in the negative control group had a IL-10 level (77.497pg/ml) above the lowest detection limit. At the end of the convalescent period, the serum IL-10 and TNF-α of the animals in negative control group and vaccine group, and the serum IL-2 of the animals in vaccine group were below the lowest detection limit. One male animal (219.304 pg/ml) and one female animal (217.369 pg/ml) in the negative control group had a IL-2 level above the lowest detection limit.

***Repeated dose toxicity test on Macaca monkey***

Objective: To evaluate the possible toxic reactions and target organs after repeated muscle administration of the inactivated SARS-CoV-2 vaccine vaccine to Macaca monkeys for 4 weeks, and the recovery of the toxic reactions after 4 weeks of discontinuation, so as to provide animal experimental data for the clinical study of the investigational vaccine .

Test design: according to the animal weight measured before grouping, 40 animals were randomly divided into 4 groups according to the sex, the low dose group, the high dose group, the negative control group and the adjuvant control group. Each group had 10 Macaca monkeys, half male and half female, respectively, with 0.5ml/300su/dose, 0.5ml/1200su/dose of investigational vaccine, 0.5ml/dose of normal saline, 0.5ml/dose of adjuvant, according to the day 0, 7 and 14. After intramuscular injection, the safety was observed until 14 days after the last administration. The detection indexes include clinical observation such as allergic reaction, injection local reaction, body weight / body temperature/ECG/blood pressure/ophthalmic examination, clinical pathological indexes (blood cell count, coagulation function, blood biochemistry, urinalysis), immunological indexes (T lymphocyte subsets, cytokines, C-reactive protein, complement, antibody), pathological examination (gross anatomy observation, histopathology).

**Results:** No death or near-death rats was observed in 4 groups, and also no clinical abnormal reaction was observed. The body weight in each group showed a normal increasing trend during the study period. No abnormalities were observed in body temperature and eye examinations. No abnormalities in blood coagulation indicators, blood biochemical indicators, urine test indicators, and T lymphocyte subsets related to drug administration were observed.

No cytokines (TNF-α, IFN-γ, IL-2, IL-4, IL-5, and IL-6) related to drug administration were observed. Within 1~2 hours (D1) after the first dose administration, the TNF-α and IFN-γ decreased in the female animals of adjuvant control group, and the TNF-α decreased in the female animals of the high dosage group; on the 3th day (D4) after the first dose administration, the TNF-α, IFN-γ, and IL-2 decreased in the female animals of the high dosage group; on the 3th day (D18) after the last dose administration, the IL-5 increased in the female animals of the adjuvant control group (adjuvant vs negative control group, 0.436 vs 0.614 pg/ml). However, the above-mentioned slightly decrease of TNF-α, IFN-γ, IL-2 has no practical significance. The IL-5 increase in the adjuvant control group has no toxicological significance, since the IL-5 level 3 days post the last dose was comparable with that before the first administration.

No clinical pathological and immunological abnormalities were observed. Microscopic observation was conducted 3 days post administration (Day 18), local granulomatous inflammation was observed in 5/6, 6/6 and 5/6 of animals in adjuvant group, low dosage group and high dosage group, respectively, with pathological changes ranging from mild to moderate. The change was considered to be local reaction induced by aluminum adjuvant, which belongs to the expected reaction induced by intramuscular injection of aluminum-containing vaccine. At the end of the 2-week recovery period (Day 29), local granulomatous inflammation was observed in 3/4, 4/4 and 4/4 of animals in adjuvant group, low dosage group and high dosage group, respectively, indicating that the local irritation reaction of administration had not yet recovered.

**Conclusion:** During the administration period and at the end of the two-week recovery period, no significant systemic toxic reactions were observed in Macaca fascicularis using 300 SU and 1200 SU, so the No Observed Adverse Effect Level (NOAEL) was considered to be 1200 SU/ Macaca fascicularis. Irritation reaction which may be related to the aluminum adjuvant was observed at the injection site, and no immune toxic reaction was observed.

***Reproductive development toxicity test on rat***

Objective: To evaluate the effect of the inactivated SARS-CoV-2 vaccine on the fertility of male and female rats, the development of pregnant / lactating female rats, embryos and fetuses, to understand the effect of the vaccine on teratogenesis and offspring development of rats, and to investigate the antibody level in the blood of embryo or offspring and to provide reference for safe drug use in different population.

Test design: according to the animal weight measured before administration, the animals were randomly divided into four groups according to the sex section, namely, the low-dose group of test sample, the high-dose group of test sample, the negative control group and the adjuvant control group. 28 male animals and 56 female animals in each group were respectively administered with 0.5mL/300SU/dose, 0.5mL/1200SU/dose of test sample, 0.5mL/dose of normal saline and 0.5mL/dose of adjuvant. Male rats were administrated 4 times before mating, respectively on the 1^st^, 8^th^ and 15^th^ day; female rats were administrated 3 times before mating, respectively on the 1^st^, 8^th^ and 15^th^ day. The male and female rats began to mate in cages one week after the drug administration, and the female rats were given drugs once on the 6^th^ day of pregnancy (GD 6) and once on the 7th day after birth (PND7). In GD20, 1/2 pregnant rats in each group were delivered by caesarean section for fetal examination (appearance, viscera and skeleton examination). The remaining 1/2 pregnant rats in the same group delivered normally until the end of lactation.

**Progress:** Both male and female rats have been completed the administration without any abnormal symptoms.

### Immunogenicity study

In order to evaluate SARS-CoV-2 Vaccine (Vero cell), Inactivated, the mice and rats were immunized intraperitoneally and intramuscularly with different doses, different adsorption methods and different immunization schedules. The blood samples were collected at different time points to determine the neutralizing antibody titer and IgG antibody titer of the serum after immunization, The immunogenicity of the vaccine and the formulation, dose and immune procedure of the vaccine are determined according to the immunogenicity results.

Study design:

- Determination of aluminum adsorption and non-aluminum adsorption processes for vaccines

Two different processes were used to prepare 1200SU/0.5mL, 600 SU/0.5mL, 300 SU/0.5mL, 150 SU/0.5mL and 1200 SU/0.5mL, 600 SU/0.5mL, 300su SU/0.5mL vaccine without aluminum adjuvant was used to immunize mice intraperitoneally, 10 mice in each group, 0.5mL in each group. Mice were immunized with one dose, and sera were collected on the 7^th^, 14^th^ and 21^st^ day after immunization. Sera of the 14th, 21^st^ and 28^th^ day were immunized with two doses, respectively, and IgG antibody titers were tested separately. At the same time, negative control animals were set. Through the comparison of antibody levels, the immunogenicity of the vaccines prepared by two different processes is compared. The specific research design is shown in the table below:

**Table2 Design of immunogenicity comparison of inactivated SARS-CoV-2 vaccines with or without aluminum adsorption**

| Dosage  (SU/0.5mL) | SARS-CoV-2 Vaccine (Vero cell), Inactivated | | | | SARS-CoV-2 Vaccine (Vero cell), Inactivated without adjuvant | | | |
| --- | --- | --- | --- | --- | --- | --- | --- | --- |
|  | Lot No. of vaccine | One dose | 0, 7  Two doses | 0, 14  Two doses | Lot No. of vaccine | One dose | 0, 7  Two doses | 0, 14  Two doses |
| 1200SU | 20200303-1 | 10 | 10 | 10 | 20200303-5 | 10 | 10 | 10 |
| 600 SU | 20200303-2 | 10 | 10 | 10 | 20200303-6 | 10 | 10 | 10 |
| 300SU | 20200303-3 | 10 | 10 | 10 | 20200303-7 | 10 | 10 | 10 |
| 150 SU | 20200303-4 | 10 | 10 | 10 | / | / | / | / |

- Determination of Immunization dose and Immunization Schedule

Mice group: the antigen content was 300SU/0.5mL, 600 SU/0.5mL, 1200 SU/0.5mL, 2400 SU/0.5mL (Batch number: 20200213-1~4). The mice were immunized with emergency immunization schedule and general immunization schedule, 10 mice/group, 0.5mL /mouse.

Rats group: the antigen content was 300SU/0.5mL, 600 SU/0.5mL, 1200 SU/0.5mL, 2400 SU/0.5mL (Batch number: 20200213-1~4). The rats were immunized with two procedures: emergency immune procedure and general immune procedure, intramuscular injection, 5 rats/group, 0.5mL/Rat. At the same time, vaccine diluent was used as negative control. Immunity and blood collection are shown in Table 3.

**Table 3 Design of Immunization dose and Immunization Schedule**

| Immunization Schedule type | Immunization Schedule | Date of blood sampling | Amount |
| --- | --- | --- | --- |
| Emergency schedule | Day 0 | Day 7, 14, 21, 28, 35, 42 | 10 Mice， 5 Rat |
|  | Day 0, Day7 | Day 14, 21, 28, 35, 42 | 10 Mice， 5 Rat |
|  | Day 0, Day 3, Day 7 | Day 7, 14, 21, 28, 35, 42 | 10 Mice， 5 Rat |
| General schedule |  |  |  |
|  | Day 0, Day 14 | Day 21, 28, 35, 42 | 10 Mice， 5 Rat |
|  | Day 0, Day 14, Day 28 | Day 35, 42 | 10 Mice， 5 Rat |

Based on the analysis of immune dose, neutralizing antibody titer and enzyme-linked antibody titer, the proposed dose for clinical trial of the vaccine was determined; meanwhile, the immune effects of one, two and three doses were compared to determine the immune procedure.

Study result:

- Determination of Aluminum adsorption and non aluminum adsorption

The vaccines containing aluminum adjuvant and the vaccine free from aluminum in mice are able to produce a certain level of novel coronavirus antibody on the 7^th^ day after initial immunization. The vaccine of 1200SU/0.5mL free from aluminum adjuvant with was the same as that of 300SU/0.5mL with aluminum adjuvant. The immunogenicity of the vaccine containing aluminum is better than that of the vaccine without aluminum.

- Determination of Immunization dosage and Immunization schedule

a. For the same immunization schedule, different dose groups immunized the same kind of animals, at the same blood sampling point, the neutralizing antibody titer was tested, and the immune dose showed a good dose-response relationship with the neutralizing antibody titer.

b. For the same dose group, different immunization procedures (one dose, two doses, three doses) immunize the same kind of animals. At the same blood sampling point, test the titer of ELISA antibody. The immune effect of mice is that the two dose and three dose schedules are not lower than the one dose schedule. The immune effect of rats is that the two dose and three dose schedules are higher than the one dose schedules. Because the interval between the three doses schedule s is short, the immune effect of the two dose procedures is equivalent to the immune effect of the three dose schedules.

c. For the same dose group, the antibody level of the two doses (0,7 days and 0,14 days) immunization schedule at different time points is one order of magnitude higher than that of the 0,7 day immunization schedule at the 21^st^ day, which indicates that the interval between the two doses shall be more than 14 days in the clinical trial.

d. For different dose groups, the neutralizing antibody levels of 1200SU and 2400SU were basically the same.

Conclusion: the dosage of aluminum adjuvant is 300 SU/dose, 600 SU/dose, 1200 SU/dose, and the Immunization Schedule is two doses.

### Study of protection against virus attack

Objective: To evaluate the protective effect of inactivated SARS-CoV-2 vaccine on animals and whether there is Antibody-Dependent Enhancement (ADE) and to provide animal test data for clinical research and use.

**Test design:** Rhesus monkeys were immunized with the vaccine with different immunization schedules and doses, and then attacked by the SARS-COV-2 seed 21~42 days after the first dose vaccination. The protection effect of the vaccine and whether there is ADE should be evaluated based on the clinical symptoms observation, serum antibody detection, and pathological examination results. The study design was shown by the following table：

**Table 4 Study design of protection effect against virus attack**

| Group | Vaccination  Schedule（Days） | Dosage | Day of attack after the first dose | Day of euthanasia  after the attack | Number of animals |
| --- | --- | --- | --- | --- | --- |
| Vaccine group  -3 doses | 0,7,14 | High dosage  (1200SU/0.5ml) | 23 | 7 | 4 |
|  |  | Medium dosage  (600SU/0.5ml) | 22 | 7 | 4 |
| Adjuvant group-  3 doses | 0,7,14 | / | 21 | 7 | 2 |
| Model (saline) group | / | / | 21 | 7 | 2 |
| Vaccine group-  2 doses | 0,14 | High dosage  (1200SU/0.5ml) | 23 | 7 | 4 |
|  |  | Medium dosage  (600SU/0.5ml) | 22 | 7 | 4 |

**Results of two-doses schedule:**

In the model group, there was no significant increase in temperature, moreover, high viral load was detected in throat swab, anal swab and lung tissues, and the lung tissue showed severe interstitial pneumonia. There was no significant difference between the adjuvant group and the model group. Compared to the model group, 2 out of 4 monkeys in medium dosage group had high fever over 40℃, and 3 out of 4 monkeys in medium dosage group was negative in throat swab virus test on Day 3, 5, 7 post virus challenge. In addition, all of the macaques were negative in throat swab virus test on Day 7 post virus challenge. All of the monkeys showed mild interstitial pneumonia, suggesting that the medium dosage vaccine had significant protective effect. Compared to the model group, the temperature of 4 monkeys in high dosage group was normal, 3 out of 4 monkeys was negative in throat swab virus test on Day 3, 5, 7 post virus challenge. In addition, all of the monkeys were negative in throat swab virus test on Day 7 post virus challenge. All of the monkeys showed mild interstitial pneumonia, suggesting that the high dosage vaccine had significant protective effect. It is noteworthy that no antibody-dependent enhancement (ADE) was observed in medium or high dosage groups . The results of antibody levels in monkeys were listed in Table 5.

**Table 5 Antibody level changes of rhesus monkeys in each group post virus challenge**

**in the study of the two-doses schedule**

|  | **Animal No.** | **0 day after administration** | **7days** | **14 days** | **21 days** | **3 days post virus challenge** | **5days** | **7days** |
| --- | --- | --- | --- | --- | --- | --- | --- | --- |
| **Medium dosage group** | **K21** | <8 | <8 | 4 | 64 | 64 | 48 | 256 |
|  | **K22** | <8 | <8 | 4 | 128 | 48 | 64 | 128 |
|  | **K23** | <8 | <8 | 6 | 48 | 32 | 96 | 1024 |
|  | **K24** | <8 | <8 | 32 | 64 | 256 | 128 | 1024 |
| **GMT** | / | / | / | 7.4 | 70.8 | 70.8 | 78.4 | 430.5 |
| **High dose group** | **K17** | <8 | <8 | 16 | 128 | 1024 | 512 | 512 |
|  | **K18** | <8 | <8 | 16 | 256 | 256 | 512 | 512 |
|  | **K19** | <8 | <8 | 4 | 96 | 512 | 1024 | 512 |
|  | **K20** | <8 | <8 | <4 | 64 | 192 | 256 | 1024 |
| **GMT** | / | / | / | 6.7 | 119.1 | 400.7 | 512.0 | 608.9 |
| **Adjuvant group** | **K9** | <8 | <8 | <4 | <4 | <4 | 4 | 8 |
|  | **K10** | <8 | <8 | <4 | <4 | <4 | <4 | <8 |
| **GMT** | / | / | / | / | / | / | / | / |
| **Model group** | **K15** | <8 | <8 | <4 | <4 | <4 | 6 | 12 |
|  | **K16** | <8 | <8 | <4 | <4 | <4 | 8 | 8 |
| **GMT** | **/** | / | / | / | / | / | 6.9 | 9.8 |

**Results of three-doses schedule:**

There was no significant increase in temperature in the model group, and no abnormalities in temperature in adjuvant group, medium dosage group and high dosage group. Animals in all groups experienced the decrease of white blood cell account and increase of lymphocyte percentage, with no significant difference among groups. The blood biochemical test results were within normal range on day 0, day 14 after the first dose vaccination, and the day of euthanasia. High level of viral load was detected in the pharyngeal swab, anal swab and lung tissue of the model group. Seven days after the virus attack, compared with the model group, the virus load in the pharyngeal swab and anal swab of medium dosage group decreased, and that of the high dosage group converted to negative. Additionally, virus load in the lung tissue of all the 3 animals in the medium dosage group and 4 animals of the high dosage group were negative 7 days after the virus attack.

Twenty one days after the first dose vaccination, animals of model group and adjuvant group were seronegative in the neutralizing antibody, and the GMT of neutralizing antibody in the medium dosage group and high dosage group was 1:61.3 and 1:400.7 respectively. Seven days after the virus attack, the GMT of neutralizing antibody in the medium dosage group and high dosage group was 1:400.7 and 1:145 respectively.

The results of antibody levels in macaques were listed in Table 6.

**Table 6 Antibody level changes of rhesus monkeys in each group post virus challenge in the study of the three-doses schedule**

|  | **Animal**  **No.** | **Day of administration**  **(Day 0)** | **Day 7** | **Day 14** | **Day 21** | **3 days after virus attack** | **5 days after virus attack** | **7 days after virus attack** |
| --- | --- | --- | --- | --- | --- | --- | --- | --- |
| **Medium dosage group** | K5 | <8 | <8 | 6 | 64 | 32 | 384 | 1024 |
|  | K6 | <8 | <8 | 4 | 24 | 32 | 64 | 512 |
|  | K7 | <8 | <8 | 48 | 384 | 128 | 512 | 768 |
|  | K8 | <8 | <8 | 6 | 24 | 32 | 64 | 64 |
| **GMT** |  | / | / | 9.1 | 61.3 | 45.3 | 168.5 | 400.7 |
| **High dosage group** | K1 | <8 | <8 | 12 | 48 | 24 | 96 | 256 |
|  | K2 | <8 | <8 | 16 | 64 | 96 | 512 | 384 |
|  | K3 | <8 | <8 | 6 | 32 | 24 | 48 | 96 |
|  | K4 | <8 | <8 | 6 | 64 | 16 | 48 | 48 |
| **GMT** |  | / | / | 9.1 | 50.1 | 30.7 | 103.2 | 145.9 |
| **Adjuvant group** | K9 | <8 | <8 | <4 | <4 | <4 | 4 | 8 |
|  | K10 | <8 | <8 | <4 | <4 | <4 | <4 | <8 |
| **GMT** |  | / | / | / | / | / | / | / |
| **Model group** | K15 | <8 | <8 | <4 | <4 | <4 | 6 | 12 |
|  | K16 | <8 | <8 | <4 | <4 | <4 | 8 | 8 |
| **GMT** |  | / | / | / | / | / | 6.9 | 9.8 |

The pathological results of some animals were shown in Figure 1-6.


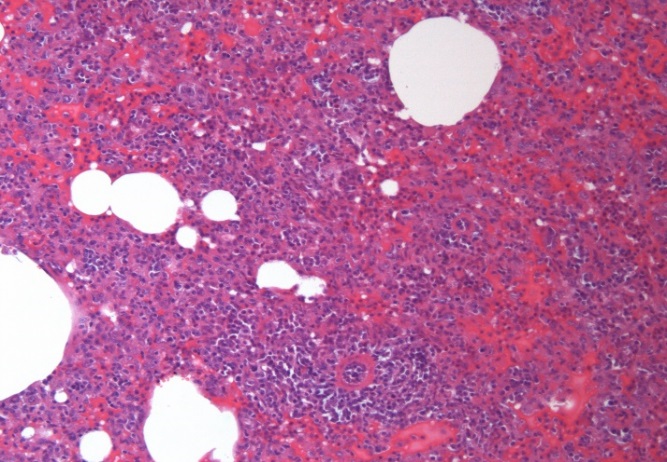

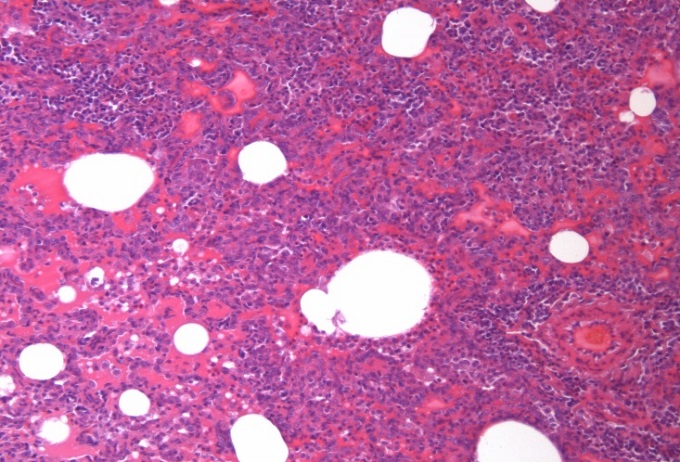


Figure 1: Model group K15 right lung accessory lobe, severe interstitial pneumonia H.E.×100

Figure 2: Adjuvant group K10 right lung medium lobe, severe interstitial pneumonia H.E.×100


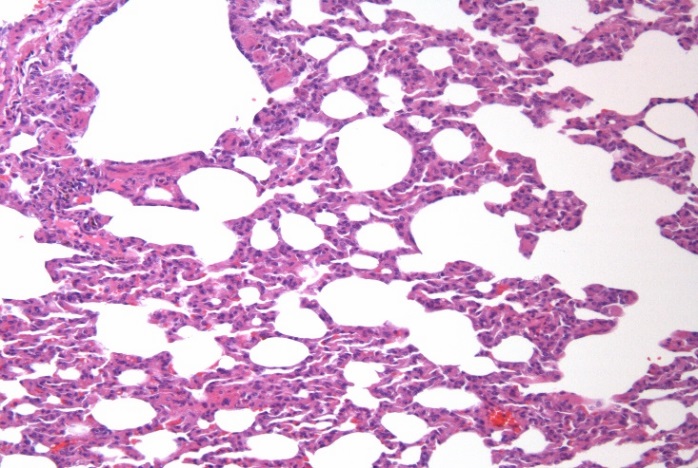

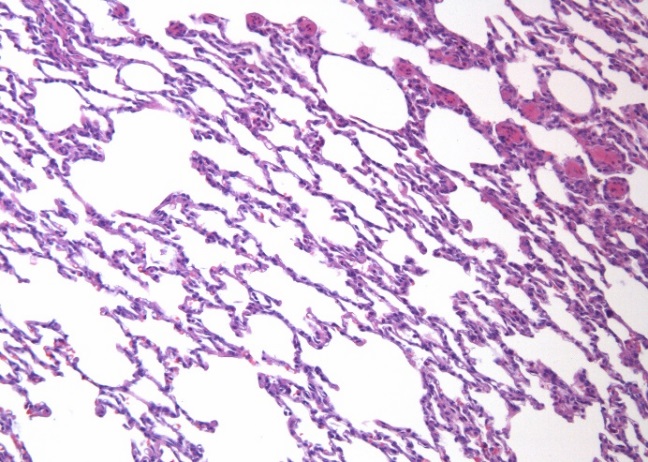


Figure 4: Medium dosage group K22 left lung upper lobe, no abnormality H.E.×100

Figure 3: Medium dosage group K21 right lung upper lobe, mild interstitial pneumonia H.E.×100


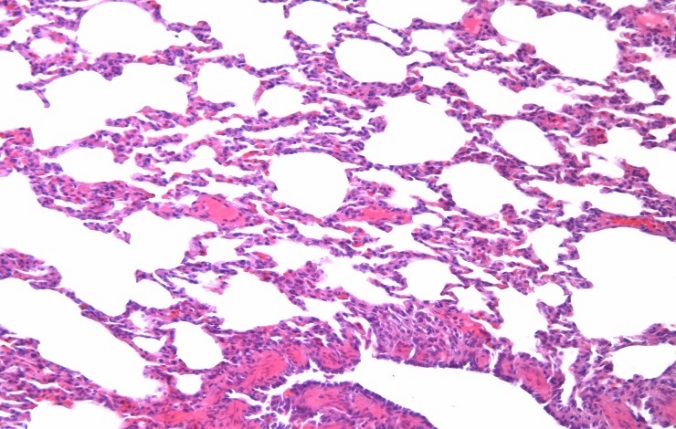

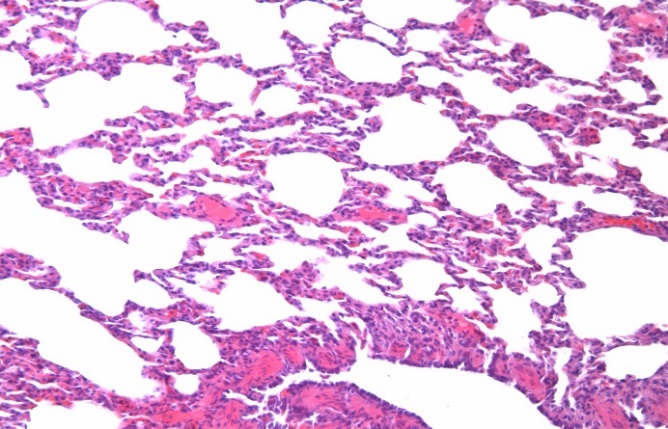


Figure 5: High dosage group K17 right lung inferior lobe, mild interstitial pneumonia H.E.×100

Figure 6: High dosage group K19 left lung inferior lobe, no abnormality H.E.×100

According to the immune protection results of the middle dose group and the high dose group, combined with the neutralizing antibody level before the attack, it is suggested that the neutralizing antibody titer of 1:48 or more can play a significant protective role.

**Conclusion:** the inactivated SARS-CoV-2 vaccine has obvious protective effect. No ADE is observed.

### Study of Cross Neutralization

The cross neutralization tests of different viruses were carried out in the sera of patients with acute stage and convalescent stage. The preliminary results showed that the overall neutralization antibody level in the sera of acute stage was not high, and a certain proportion of neutralization antibody in the sera was negative; the overall neutralization antibody level in the serum of convalescent stage was higher than that in the serum of acute stage, and the preliminary data showed that 100% neutralization antibody in the serum of convalescent stage was positive, these data results can indicate that neutralizing antibody plays a protective role in the recovery of patients.

The neutralizing antibody titer of the animal serum immunized with the vaccine was also tested. The results showed that the neutralizing antibody titer of the vaccine could be produced by using the non vaccine strain virus, and it had a good dose-response relationship with different immune doses and immune procedures.

Cross neutralization tests will be continued.

## Clinical trial of SARS-CoV-2 vaccine in healthy adults

In order to evaluate the safety, tolerability and preliminary immunogenicity of different dosage vaccine administered at different immunization schedules in adults, a randomized, double-blind and placebo-controlled Phase I/II clinical trial was carried out in Suining County, Jiangsu Province in April 2020. Till now, 144 subjects in phase I (143 subjects completed the first dose of vaccination) and 600 subjects in phase II (all completed the first dose of vaccination) have been enrolled.

Till now, safety and immunogenicity results for emergency schedule (Day 0,14) were reported from 72 adults in Phase I and 300 adults in Phase II. The routine schedule (Day 0,28) is still ongoing.

For safety in Phase I, the incidence rates of solicited adverse reactions in high dosage, medium dosage and placebo group were 37.50%, 25.00% and 8.33%, respectively. In Phase II, the incidence rates of adverse reactions in high dosage, medium dosage and placebo group were 37.50%, 25.00% and 8.33%, respectively. The incidence rates of adverse reactions in two vaccine groups were higher than that in placebo group, however, no statistically significant difference was found between the medium and high dosage group. The adverse reactions were mainly Grade 1, and no serious adverse reaction occurred. For immunogenicity, the seroconversion rate in Phase I in high dosage, medium dosage and placebo group were 50.00%, 45.83% and 0.00%, respectively. The GMT in high dosage, medium dosage and placebo group were 7.7, 5.6 and 2.0, respectively. The seropositive rate of IgG antibody in high dosage, medium dosage and placebo group were 95.83%, 75.00% and 8.33%, respectively. While the IgM were 45.83%, 20.83% and 0.00%, respectively. Participants in the placebo group failed to induced immune response. In Phase II, the seroconversion rate in high dosage, medium dosage and placebo group were 98.32%, 92.37% and 3.33%, respectively. The GMT in high dosage, medium dosage and placebo group were 34.5, 27.6 and 2.3, respectively. The GMT in high dosage and medium dosage group were comparable. For cellular immunity, no positive specific T cell response was observed before vaccination in 72 participants. 14 days post first vaccination, no positive specific T cell response was observed in medium dosage group. The positive rate in high dosage group was 12.5%. 14 days post second vaccination, the positive rates in high dosage, medium dosage and placebo group were 20.83%, 45.83% and 8.33%, respectively. All the data did not provide evidence on the dose-response relationship. Based on these results, we did not observe obvious cellular immunity induced by SARS-CoV-2 Vaccine, and further study should be conducted.

Conclusion: In conclusion, all the date from Phase I/II clinical trial indicated favorable safety and immunogenicity with a two-dose schedule of SARS-CoV-2 Vaccine. The GMT in high dosage and medium dosage group were comparable. Based on the results, we did not observe obvious cellular immunity induced by SARS-CoV-2 Vaccine, and further study should be conducted. No significant changes of inflammatory factors were observed, indicating a small risk of immunopathology induced by SARS-CoV-2 Vaccine.

## Product features

### Preparation process and Formula of vaccine

SARS-CoV-2 Vaccine（Vero Cell），Inactivated is prepared by novel coronavirus (CZ02 Strain) inoculated in African green monkey kidney cells (Vero Cells), after culture, harvesting virus solution, inactivating virus, concentration, purification and aluminum hydroxide adsorption. It is a milky white suspension liquid, which can be layered due to precipitation and easily dispersed. The novel coronavirus (SARS-CoV-2) is the main component. The excipients are aluminum hydroxide, disodium hydrogen phosphate, sodium dihydrogen phosphate, sodium chloride, etc., It is free from preservatives. Packaging for vials or pre filled syringes is 0.5mL for each container. The novel coronavirus can induced novel coronavirus immunity after inoculation, and it can be used to prevent the disease caused by the new coronavirus infection.

The investigational vaccine is produced by Sinovac Research & Development Co., Ltd. and tested by National Institute for Food and Drug Control as per *Manufacturing and Quality Control Requirements of SARS-CoV-2 Vaccine（Vero Cell），Inactivated (Draft Version)* and complied. The vaccine is a injection with the strength of 0.5mL/container.

### Placebo vaccine

Formulation, dose, route of administration: Aluminium hydroxide, disodium hydrogen phosphate, sodium dihydrogen phosphate, sodium chloride 0.5mL/dose, intramuscular injection, two doses given 14 days apart.

The vaccine is reviewed and retested by National Institutes for Food and Drug Control, China and the results complied with all requirements.

### Storage and Transportation

It shall be stored and transported at 2~8℃, prevent from light.

### Dosage and administration:

Each prefilled syringe of the vaccine contains 600SU of SARS-CoV-2 virus antigen. The routine immunization procedure is 2 doses at 14-day interval, each inoculation dose is 0.5 mL. The recommended site of administration is deltoid of upper arm by intramuscular injection

### Information of Investigational vaccine

The vaccine is developed by Sinovac Research & Development Co., Ltd. It is a preparation made from novel coronavirus (strain CZ02) grown in African green monkey kidney cell cultures (Vero Cell). After cultivation, harvest, the virus suspension is inactivated, concentrated, purification, and adsorbed by aluminium hydroxide. It contains inactivated SARS-CoV-2 Virus, aluminium hydroxide, disodium hydrogen phosphate, sodium dihydrogen phosphate, sodium chloride Trade name: CoronaVac 0.5mL/dose containing 600SU of SARS-CoV-2 virus antigen. Intramuscular injection, two doses given 1 month apart.

### Inactivation process

The inactivation process is described as β-propiolactone is added in the virus harvest fluid at a ratio of 1:4000 and inactivated at 2-8°C for 12-24 hours. β-propiolactone and formaldehyde solution are dispensed with intermediate from the primary inactivation at a ratio of 1:4000 for the secondary inactivation at 2-8°C for 2-8 hours, inactivated SARS-CoV-2 fluid is obtained.

Following the determined inactivation process parameter to inactivate 9 lots of inactivation fluid and perform inactivation verification. The results show that following the determined inactivation process, the SARS-CoV-2 virus can be inactivated completely, which demonstrates that the inactivation process is stable.

# ObjectIves

**Primary objective:**

- To evaluate the efficacy of a two-dose regimen of SARS-COV-2 (Vero Cell) vaccine against RT-PCR confirmed symptomatic COVID-19.

**Secondary Objectives:**

- To evaluate efficacy of , at least, one dose of SARS-COV-2 (Vero Cell) vaccine agianst RT-PCR confirmed symptomatic COVID-19
- To evaluate efficacy of two-dose regimen of SARS-COV-2 (Vero Cell) vaccine against severe COVID-19 cases and rates for hospitalization and death
- To evaluate efficacy of a two-dose regimen of SARS-COV-2 (Vero Cell) vaccine against RT-PCR confirmed SARS-CoV-2 infection
- To evaluate the safety of SARS-CoV-2 (Vero Cell) vaccine (inactivated)
- To evaluate the immunogenicity of SARS-CoV-2 (Vero Cell) vaccine
- To evaluate the duration of immunogenicity of SARS-CoV-2 (Vero Cell) (inactivated) vaccine up to 120 days

**Primary endpoint:**

- The protection rate of a two dose of SARS-CoV-2 (Vero Cell) vaccine against RT-PCR confirmed symptomatic COVID-19 two weeks after the second dose of vaccination.

**Secondary endpoints:**

- The protection rate of, at least, one dose of SARS-CoV-2 (Vero Cell) vaccine against RT-PCR confirmed symptomatic COVID-19 two weeks after the last dose vaccination.
- The protection rate of a two dose of SARS-CoV-2 (Vero Cell) vaccine against rates of hospitalization, disease severity/and death two weeks after the second dose of vaccination.
- The protection rate of a two dose of SARS-CoV-2 (Vero Cell) vaccine against RT-PCR confirmed SARS-CoV-2 infection two weeks after the second dose of vaccination.
- The incidence of adverse reactions from the day of first vaccination to 28 days after the second dose of vaccination.
- The incidence of adverse reactions within 7 days after each dose of vaccination
- The incidence of SAEs from the first vaccination to one year after the second dose vaccination.
- The seroconversion rate, seropositivity rate, GMT and GMI of neutralizing antibody and IgG 14 days after each dose vaccination.
- The seroconversion rate, seropositive rate, GMT and GMI of neutralizing antibody and IgG 28 days after the second dose vaccination

# Study desIgn/type

This will be a case driven Phase III, individually randomized, double blind, placebo-controlled clinical trial. The manufacturer has also agreed with Bangladesh and Brazil to start Phase III studies with SARS-CoV-2 (Vero Cell) vaccine (inactive). Studies designed in these countries are in the process of approval.

### Randomization and Unblinding

Eligible subjects will be randomized at their Study Visit Day 0 using a ratio of 2:1 (SARS-Cov-2 Vaccine: placebo) to each study group using Omega CRO IWRS (Interactive Web Response System). Unblinding will also be managed by using the same system. The system customizes the randomization algorithm. When a subject is enrolled, email from the system notifies instantly. After enrolment in the study, each participant will be randomly assigned to either of the two treatment arms at a ratio of 1:1 at high risk population and 2:1 at normal risk population. Each enrolled participant will be assigned a code receive the treatment labelled with the code. This code will be captured in the participants source documents and electronic data base system.

The blinding may be broken by the Investigator in this study in the event of a medical emergency in which knowledge of the identity of the study vaccine is critical to the management of the subject’s immediate. If deemed necessary to break the blinding for a study subject, DSMB is to be contacted to obtain concurrence. If it is not possible to contact DSMB beforehand, he or she should be contacted as soon as possible after breaking the blinding for a subject. Details regarding the emergency unblinding will be documented in Omega IWRS. Any subject whose blinding has been broken will continue to participate in subsequent visits.

### Sample Size

The study is planned to be carried out with two separate cohorts. The first cohort (K-1) includes healthcare professionals working in healthcare units; second cohort (K-2) includes healthy population at normal risk for COVID-19 disease. Primarily, high risk healthcare professionals will be included to the study, they will be randomized 1:1 ratio and they will be followed and K-2 cohort will be initiated after evaluation of interim safety report by Data and Safety Monitoring Board. Both cohorts will be followed up for RT-PCR confirmed symptomatic COVID-19 disease. If the clinical efficacy of vaccine is shown in K-1 or K-2, subjects randomized to placebo arm will also be vaccinated.

In K-1 cohort, 588 subjects should be included to both arms with the assumption of the risk of infected with COVID-19 will be 5% for placebo arm and %2 for vaccine arm in the high risk population. Considering 10% of drop-out rate and 5% of seropositive or PCR positive at baseline, 680 subjects should be screened at both arms of cohort 1.

Group sample sizes of 7545 SARS-CoV-2 vaccine and 3773 placebo produce a two-sided 95% confidence interval for the difference in population proportions with a width that is equal to %1,0 when the estimated incidence rate for vaccinated group is %1,0 and the estimated incidence rate for placebo group is %2,0. Drop out rate is assumed as 10% and with 5% either seropositive or PCR positive at baseline, it is needed to be enrolled 13.000 participants totally in both cohorts. The remaining 11.640 subjects will be screened in the cohort 2 and eligible subjects will be randomized in 2:1 ratio. Distribution of subjects to both cohorts is shown in below figure.

Subjects in placebo arm will be administered by SARS-CoV-2 vaccine if its efficacy can be demonstrated with an interim analysis performed with 40 confirmed COVID-19 cases. The rate of difference of number of cases in vaccine arm and placebo arm reaches 60%, placebo arm will be vaccinated. Since the study will be initiated with healtcare professionals at high risk, 5% of placebo arm (29 subjects) and 2% of vaccinated arm (11 subjects) are estimated to be infected and this will demonstrate the clinical efficacy. If those rates can not be obtained in Cohort 1, the study will be continued with Cohort 2 as defined above. If those rate can be obtained in Cohort 1, placebo arm in Cohort 2 will be removed.

In a blinded analysis, if less than 50 cases occurred during 3 months of follow-up after the last subject enrollment, the DSMB will compare the actual primary endpoint case count to pre-established criteria to determine if a sample size increase is necessary.


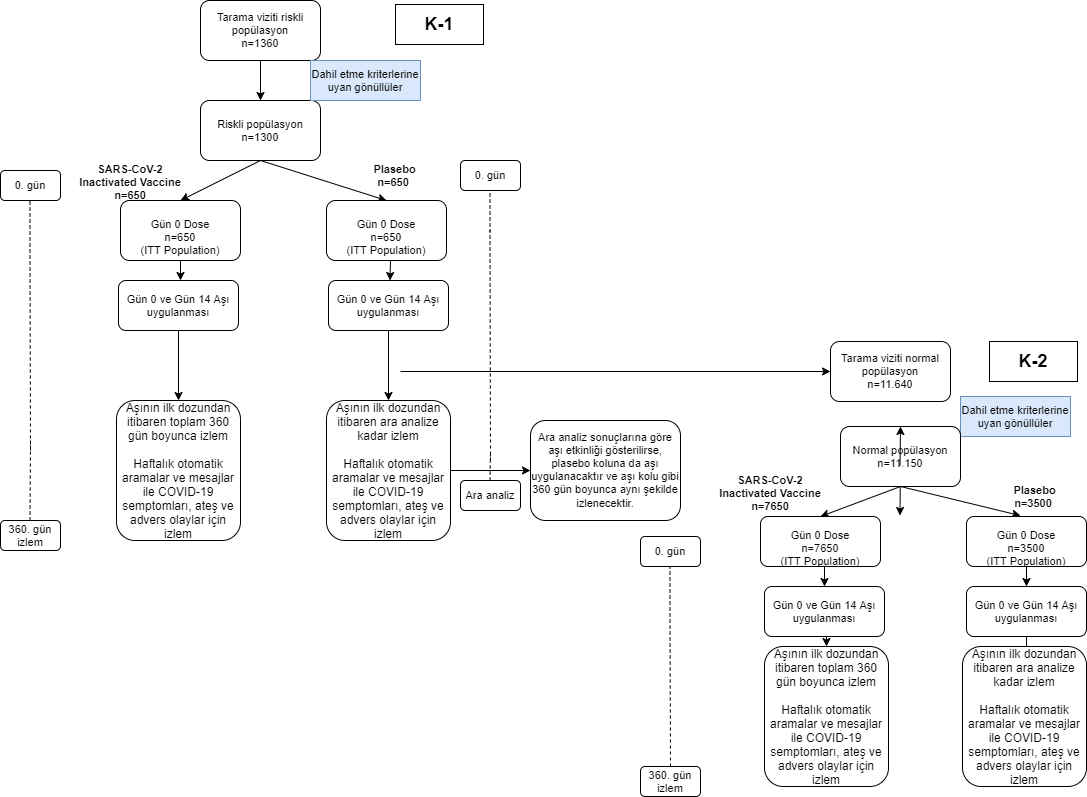


**Figure 1. Distribution of subjects to Cohort 1 and Cohort 2**

### Eligibility criteria

**Inclusion criteria:**

Inclusion Criteria:

1. 18-59 years of age (including 18 and 59 years of ages),
2. For only K1 cohort, health care workers such as medical doctor, nurse, ward boy, cleaner, hospital technician, administrative personnel who work in any department of a healthcare unit.
3. Signed informed consent

Exclusion criteria:

1. Previously PCR positive for COVID-19
2. IgG or IgM is positive
3. For females: Pregnancy (confirmed by positive beta-hCG test), breastfeeding or intent to engage in sexual relations with reproductive intent without use of birth control methods in the three months following vaccination
4. Known allergy to components of the study vaccine or control
5. Use of immunosuppressant therapy regimens within the six months prior to enrollment in the study or planned use within the two years following enrollment. Immunosuppressant therapy regimens include: antineoplastic chemotherapy, radiation therapy and immunosuppressants to induce transplant tolerance, among others
6. Use of immunosuppressive doses of corticosteroids within the three months prior to the enrollment in the study and planned use of immunosuppressive doses of corticoids within the three months following enrollment in the study. Immunosuppressive doses of corticosteroids will be considered the equivalent prednisone 20 mg/day for adults, for longer than one week. Continued use of topical or nasal corticosteroids is not considered an immunosuppressant
7. History of asplenia
8. History of bleeding disorder (e.g. factor deficiency, coagulopathy or platelet disorder), or prior history of significant bleeding or bruising following IM injections or venipuncture
9. Any alcohol or drug abuse over the 12 months prior to enrollment in the study that has caused medical, professional or family problems, indicated by clinical history
10. Administration of immunoglobulins and/or any blood products within the three months preceding the planned administration of the vaccine candidate
11. Participation in another clinical trial with an investigational product in the six months prior to enrollment in the study or planned participation in another clinical trial within the two years following enrollment
12. Received live attenuated virus vaccine 14 days prior to enrollment in the study
13. Inactivated vaccine or sub unit vaccine 7 days prior to enrollment in the study
14. Fever (oral temperature >37.2℃, axillary temperature will not be accepted) within the past 24 hours
15. Any other condition that, in the opinion of the principal investigator or his/her representative physician, could put the safety/rights of potential participants at risk or prevent them from complying with this protocol.
16. Any confirmed or suspected autoimmune disease or immunodeficiency disease, including human immunodeficiency virus (HIV) infection.

Exclusion criteria for the second dose

1. If one of the following adverse events (AE) occurs, it is forbidden to continue vaccination, but other research steps can be continued other steps according to the researcher' judgement;
   1. During the study, the vaccine similar with the experimental vaccine was used；
   2. Any serious adverse reactions are related with the inoculated trial vaccine；
   3. Severe anaphylaxis or hypersensitivity after vaccination (including urticaria / rash within 30 minutes after vaccination)；
   4. Any confirmed or suspected autoimmune disease or immunodeficiency disease, including human immunodeficiency virus (HIV) infection.
2. If one of the following adverse events occurs, the researcher will judge whether to vaccinate; ;
   1. Acute or new chronic disease after vaccination；
   2. Other reactions (including severe pain, severe swelling, severe limitation of movement, persistent high fever, severe headache or other systemic or local reactions) were judged by the researchers
3. If one of the following events occurs, the vaccination can be postponed within the time frame specified in the protocol;
   1. Acute diseases occur during vaccination (acute disease means moderate or severe disease with or without fever)；
   2. Oral temperature during vaccination >37.2℃；
   3. Vaccinated with sub unit vaccine or inactivated vaccine within 7 days, immunized with live attenuated vaccine within 14 days;
   4. According to the investigator's judgment, the subject has any other factors that affect vaccination.

### Withdrawal of Participation

According to the principles of the Declaration of Helsinki and any other applicable regulations, a participant has the right to withdraw from the study at any time and for any reason, and is not obliged to give his or her reasons for doing so. The investigator may withdraw a participant at any time in the interests of the participant’s health and well-being. The participation may be withdrawn for any of the following reasons:

- Administrative decision by the investigator.
- Ineligibility (either arising during the study or retrospectively, having been overlooked at screening)
- Significant protocol deviation.
- Participant’s non-compliance with study requirements
- An AE, which requires discontinuation of the study involvement or results in inability to continue to comply with study procedures.
- For females, positive by the urine pregnancy test at day of dosing with first or second dose of vaccine.

The reason for withdrawal will be recorded in the CRF. If withdrawal is due to an AE, appropriate follow-up visits or medical care will be arranged, with the agreement of the participant, until the AE has resolved. If a participant withdraws from the study, data and blood, nasopharyngeal samples collected before their withdrawal will still be used in the analysis. Storage of blood/ nasal/nasopharyngeal samples will continue unless the participant specifically requests otherwise.

### Termination of the Study

The study will be terminated if the followings occur:

1. More than 15% of the subjects have grade 3 and above adverse events related to vaccination, including local reaction, systemic reaction and vital signs
2. Observation of disease enhancement after vaccination.

### Study Method

All potential participants of the study will not be admitted to COVID-19 clinics of the study sites. Separate sections will be created for study participants. This will ensure that patients with COVID-19 and participants are not in the same place at all.

**Vizit 0 Day -3: Screening visit: Informed consent, IgG and IgM antibody, nucleic acid screening, determining eligible subjects**

- **Consent process:** All potential participants will have a screening visit for baseline PCR test for COVID-19. During this screening period all study procedures will be discussed with the participant, informed consent will be obtained and documented, and criteria for inclusion and exclusion will be evaluated. The participant will provide his/ her signature on written informed consent for their participation in the study before any study related procedure or eligibility assessment is performed. The participant will be allocated a unique screening number after written informed consent is obtained.
- Check inclusion and exclusion criteria
- Urine pregnancy test for women of reproductive age
- Any other illnesses – like diabetes, hypertension, cardiac illness etc will be recorded
- Nasopharyngeal swab will be collected for laboratory test for SARS-CoV-2 virus detection. The procedure for laboratory detection of the virus is described in section under ‘Covid-19 Drug Trial Laboratory Protocol’.
- A participant ID (PID) will be assigned
- Blood sample: Approximately 5.0 ml blood will be collected from all participants

**Visit 1 Day 0: Randomization, 1st dose of SARS-CoV-2 Inactivated vaccine or placebo, safety observation**

A subgroup of consequent 1000 vaccinated participants’ blood will be taken for the analysis of neutralizing antibody and immunogenicity. Participants of this subgroup will be included from Ankara since the bloods for T-cell activation tests should be delivered to laboratory in maximum 4 hours. The blood samples will be taken from both placebo and vaccinated groups, in order not to break the blind, until reaching the 1000 vaccinated samples. 600 blood samples will be analysed for neutralizing antibody, IgG in China by manufacturer and all vaccinated 1000 blood samples will be analysed in Ankara University Faculty of Veterinary, Department of Virology.

- Demographic data will be collected
- Vital signs, height, weight will be measured
- Medical history will be collected
- Physical examination will be done
- Information on concomitant medication will be gathered
- Randomization will be done
- Study vaccine or placebo will be administered according to randomization
- Observation for 30 minutes following vaccination.
- After 30 minutes injection site will be inspected

Participants will be requested to measure their body temperature daily and note to diary card for 15 days after second dose of vaccine, along with the emergency 24 hour telephone number to contact 24/7 Call Centre/ the study physician if needed. Participants will be instructed on how to fill up and self-assess the severity of these AEs. There will also be space on the diary card to self-document unsolicited AEs, and whether medication was taken to relieve the symptoms for 1 month after each dose. AE and COVID-19 symptoms will be recorded to diary cards when they developed during one-year follow-up period. Our staff will follow up daily over phone during this period. Diary cards will collect information on the timing and severity of the following solicited AEs:

**Table 5. Solicited AEs as collected on post vaccination diary cards for reactogenicity subset**

| **Local solicited AEs** | **Systemic solicited AEs** |
| --- | --- |
| Pain | Fever |
| Rash | Diarrhea |
| Redness | Decreased Appetite |
| Pruritus | Joint pains |
| Swelling | Muscle pains |
| Induration | Fatigue |
|  | Headache |
|  | Cough |
|  | Nausea |
|  | Pruritus |
|  | Allergic reaction |
|  | Skin rash |
|  | Vomiting |

**Visit 2 Day 14 (+5 days)-Receive 2nd dose of SARS-CoV-2 Inactivated vaccine or placebo, antibody test, safety observation**

- For the analysis of neutralizing antibody and immunogenicity; blood samples will be taken from the same participants whose blood taken in Visit 1 Day 0.
- Physical examination
- Vital signs will be measured
- Evaluation for exclusion criteria for second dose
- Study vaccine or placebo will be administered according to randomization
- Collect Diary cards for temperature measurement, COVID-19 symptoms and safety observation
- Observation for 30 minutes following vaccination.
- After 30 minutes injection site will be inspected

If a study participant is unable to receive the second dose of the vaccine for any reason, he / she will be invited to remain in the study for the following visits and for surveillance of COVID-19 cases.

**Visit 3 Day 28 (+5 days)-Antibody test (for 1000 randomized subgroup), safety observation, follow-up**

- For the analysis of neutralizing antibody and immunogenicity; blood samples will be taken from the same participants whose blood taken in Visit 1 Day 0. Also 1000 samples which are obtained from the same participants will be analysed for T cell activation and percentage of T cell proliferation in Hacettepe University, Faculty of Medicine, Basic Oncology, Immunology Laboratory.
- Collect Diary cards for temperature measurement, COVID-19 symptoms and safety observation
- Information on concomitant medication will be gathered

**Visit 4 Day 42 (+7 days): Antibody test , safety observation, follow-up (for 1000 randomized subgroup)**

Same procedure with Visit 3 Day 28. Diary cards will be obtained on 28^th^ day at last.

**Visit 5 Day 194 (±20 day): Antibody test , safety observation, follow-up (for 1000 randomized subgroup)**

- For the analysis of neutralizing antibody and immunogenicity; blood samples will be taken from the same participants whose blood taken in Visit 1 Day 0.

**Unscheduled Visits:**

If symptoms are developed, nasopharyngeal swab will be collected for laboratory test for SARS-CoV-2 virus detection and RT-PCR will be performed.

Safety information and COVID 19 symptoms of subjects will be collected if subjects inform any adverse event and symptoms on those visits

### Follow-up Contacts

The purpose of the follow-up contacts is to verify the occurrence of adverse events and cases of COVID-19 among the participants. These contacts may be made automatic telephone calls or in person, at the discretion of the study team and the participant who will inform the team about the contact forms they prefer. Contact can also be made by home visit or the research center, if necessary.

Contacts will be made in a week after each vaccination and thereafter every week for the first 13 weeks after vaccination and every two weeks until 12 month-follow-up period completed.

Any contact made, or attempted contact with the participant, must be registered in the IWRS system of Omega Araştırma.

Procedures:

1. Actively check for suspected cases of COVID-19;
2. Check for unsolicited adverse events;

### Visits for placebo arm

Subjects in placebo arm will be administered by SARS-CoV-2 vaccine if its efficacy can be demonstrated with an interim analysis. After vaccination, they will be followed up for additional 12 months as explained above.

### Procedures in The Event of Fever and Suspicion of COVID-19

The participant should be constantly advised to seek out the study team whenever he or she has a fever or other symptoms related to COVID-19, to assess whether it is a possible case. The study team is responsible for establishing a routine that allows this participant to be evaluated as soon as possible as soon as he contacts the team.

In all possible cases, clinical samples for the detection of SARS-CoV-2 and a blood sample for serology (8.5 mL) will always be collected. In addition, a study physician must perform and record the participant's clinical evaluation. It should be noted that this assessment can be made from the second day of symptoms. The clinical presentation of COVID-19 can vary widely and can occur concurrently with other diseases, which justifies the recommendation to collect laboratory samples to check COVID-19 in all possible cases, so that the diagnosis of COVID-19. All possible cases must be followed up to the resolution of all symptoms and the duration and severity of each of the signs and symptoms must be documented. Table 9 can be used to classify the severity of these signs and symptoms (see section 13). Particular attention should be paid to the early detection of alarm signs and symptoms associated with severe COVID-19 in order to offer treatment in a timely and decrease the severity of clinical complications. Clinical case management will follow the guidelines of Turkish Ministry of Health according to clinical severity. Hospitalized cases must be monitored daily to verify their evolution according to the scale of clinical progression. Outpatient cases will only have the maximum severity and duration of symptoms recorded.

Local health authorities can request sample collection for the diagnosis of COVID-19. In this case, another battery of samples will be collected, different from the samples needed for the study. If another battery of samples is collected to meet local legal requirements, the results of these tests should be noted if the participant agrees to provide this information. An aliquot of samples collected for the study may also be provided for local analysis. In any case, the results of the study will be available for participants to take preventive action and report this data to local health authorities.

The performance of the tests of participants with a possible case of COVID-19 varies according to the evaluation day after the onset of symptoms. If the suspicion persists after obtaining a negative test result, a new clinical sample should be collected with an interval of at least two days. Oxygen saturation will be measured in all cases to complement the clinical evaluation. A blood sample (8.5 mL) should also be collected to assess serological response parameters

### COVID-19 CASE DEFINITIONS

The definition of case caused by the 2019 SARS-CoV-2 (COVID-19) that will be used in this study will be that stated by the FDA guides [26], as follows:

Anyone who has at least one of the following symptoms for two days or more should be tested to detect SARS-CoV-2 nucleic acid in a clinical sample:

- Fever or chills;
- Cough;
- Shortness of breath or difficulty in breathing;
- Fatigue;
- Muscle or body pain;
- Headache;
- Loss of smell or new taste;
- Sore throat;
- nasal congestion or runny nose;
- Nausea or vomiting;
- Diarrhea.

The definition of symptoms is subjective and depends on what each participant considers abnormal for their routine condition. It is recommended to evaluate all participants between the second and the seventh day of presentation of these symptoms, although collection is allowed until the fourteenth day after the onset of symptoms.

**Laboratory criteria**

Detection of SARS-CoV-2 nucleic acid in a clinical sample.

**Classification of cases**

*Possible:*

Anyone who meets the clinical criteria.

*Confirmed case:*

Anyone who meets the laboratory criteria.

*Discarded case:*

A discarded case is a possible COVID-19 case that had two negative RT-PCR tests for detection of SARS-CoV-2 nucleic acid with at least two days between them. For the second test the sample collection period for nucleic acid detection must be repeated within 14 days after the onset of symptoms, preferably within seven days. If the second collection exceeds 14 days, a serological sample will be evaluated until the 28th day after the onset of symptoms to assess possible recent exposure.

*COVID severe case:*

Refers to a laboratory confirmed case of SARS-CoV-2 infection that has one or more of the following conditions:

- Clinical signs at rest indicating severe systemic disease (respiratory rate ≥ 30 per minute, heart rate ≥ 125 per minute, oxygen saturation ≤ 93% at room temperature at sea level or PaO2 / FiO2 <300 mm Hg);
- Respiratory failure (defined as the need for high-flow supplemental oxygen, non-invasive ventilation, mechanical ventilation or extracorporeal oxygenation);
- Evidence of shock (Systolic BP <90 mm Hg, Diastolic BP <60 mm Hg, or need for vasopressors);
- Major acute renal, hepatic or neurological dysfunction;
- Admission to the Intensive Care Unit;
- Death.

### Clinical progression scale for SARS-CoV-2 infection

All cases of SARS-CoV2 infection will be classified according to the scale of clinical progression proposed by the World Health Organization [41] according to Table 9. The evaluation of hospitalized cases (score 4 or higher) will be done daily until the resolution of symptoms. In non-hospitalized cases, the maximum score and duration of symptoms will be recorded (score 1-3).

**Table 6. Scale of clinical progression of SARS-CoV-2 infection. Adapted from a proposal by the World Health Organization**

| Score Description |  |
| --- | --- |
| Uninfected, viral RNA not detected | 0 |
| Asymptomatic, viral RNA detected | 1 |
| Symptomatic, independent | 2 |
| Symptomatic, need help | 3 |
| Hospitalized *, without oxygen | 4 |
| Hospitalized, supplemental oxygen by mask or nasal cannula | 5 |
| Hospitalized, oxygen by non-invasive or high flow ventilation | 6 |
| Intubation and mechanical ventilation, P02 / FiO2 ≥150 or SpO2 / FiO2 ≥ 200 | 7 |
| Mechanical ventilation P02 / FiO2 <150 (SpO2 / FiO2 <200) or vasopressors | 8 |
| Mechanical ventilation P02 / FiO2 <150 (SpO2 / FiO2 <200) and vasopressors, dialysis or extracorporeal oxygenation | 9 |
| Death | 10 |

** If hospitalization is for isolation only, record the status with an outpatient*

### Procedure in The Event of Loss of Visit or Contact

If the volunteer does not attend any of the scheduled visits or it is not possible to establish the scheduled contact within the time set for the window, the research team will continue to try to establish contact with the participant to check if there were any adverse events that prevented the participant from going to visit or contact the contact and check if there is any case of fever. When contact is reestablished with the participant who missed a scheduled visit, an extra visit can be scheduled by collecting samples corresponding to the closest visit. After this visit, the follow-up will return to the routine established in the study schedule. Attempts to contact must be documented and must be carried out at least four weeks apart. Study teams will cease contact attempts in the absence of any effective contact after a 13-week trial period.

In case of suspected adverse event, when contact is resumed, the participant may be summoned for evaluation on an extra visit. If there is no safety concern, the participant can be assessed on the next scheduled visit.

**Table 6. Schedule of Study Procedures (whole group)**

| **Visit** | **Visit 0** | **Visit 1** | **Visit 2** | **Visit 3** | **Unscheduled Visits***** |
| --- | --- | --- | --- | --- | --- |
|  | **Screening** | **Day 0** | **Day 14** | **Day 28** | **Weekly phone visits** |
| Preliminary information | **X** |  |  |  |  |
| Informed consent | **X** |  |  |  |  |
| Screening for inc/excl. criteria | **X** |  |  |  |  |
| Physical Examination |  | **X** | **X** |  |  |
| Pregnancy Test |  | **X** | **X** |  |  |
| Total antikor (IgM+ IgG) testi | **X** |  |  |  |  |
| RT-PCR***** | **X** |  |  |  |  |
| Randomization |  | **X** |  |  |  |
| Vaccine administration |  | **X** | **X** |  |  |
| Collecting diary cards for safety observation and COVID-19 Symptoms |  |  | **X** | **X** |  |
| **COVID-19 Symptoms Follow-up by telephone visits |  |  |  |  | **X** |
| AdverseEvent and SAE Follow-up |  | **X** | **X** | **X** | **X** |
| Recording of concomitant medication |  | **X** | **X** | **X** | **X** |

* RT-PCR will be performed if any symptoms are developed during follow-up period

**Contacts will be made in a week after each vaccination and thereafter every week for the first 13 weeks after vaccination and every two weeks until 12 month-follow-up period completed

***Visits will be performed if any symptoms are developed

**Table 7. Schedule of Study Procedures for Subgroup of Neutralizing Antibody and Immunogenicity**

| **Visit** | **Visit 0** | **Visit 1** | **Visit 2** | **Visit 3** | **Visit 4** | **Visit 5** | **Unscheduled Visits***** |
| --- | --- | --- | --- | --- | --- | --- | --- |
|  | **Screening** | **Day 0** | **Day 14** | **Day 28** | **Day 42** | **Day 194** | **Weekly phone visits** |
| Preliminary information | **X** |  |  |  |  |  |  |
| Informed consent | **X** |  |  |  |  |  |  |
| Screening for inc/excl. criteria | **X** |  |  |  |  |  |  |
| Physical Examination |  | **X** | **X** |  |  |  |  |
| Pregnancy Test |  | **X** | **X** |  |  |  |  |
| Total antikor (IgM+ IgG) testi | **X** |  |  |  |  |  |  |
| RT-PCR***** | **X** |  |  |  |  |  |  |
| Randomization |  | **X** |  |  |  |  |  |
| Vaccine administration |  | **X** | **X** |  |  |  |  |
| Neutralizing antibody test (for 1000 randomized subjects-subgroup) |  | **X** | **X** | **X** | **X** | **X** |  |
| IgG test (for 1000 randomized subjects-subgroup) |  | **X** | **X** | **X** | **X** | **X** |  |
| T-Cell Activation (for 1000 randomized subjects-subgroup) |  |  |  | **X** | **X** |  |  |
| Collecting diary cards for safety observation and COVID-19 Symptoms |  |  | **X** | **X** |  |  |  |
| **COVID-19 Symptoms Follow-up by telephone visits |  |  |  |  | **X** | **X** | **X** |
| AdverseEvent and SAE Follow-up |  | **X** | **X** | **X** | **X** | **X** | **X** |
| Recording of concomitant medication |  | **X** | **X** | **X** | **X** | **X** | **X** |

* RT-PCR will be performed if any symptoms are developed during follow-up period

**Contacts will be made in a week after each vaccination and thereafter every week for the first 13 weeks after vaccination and every two weeks until 12 month-follow-up period completed

***Visits will be performed if any symptoms are developed

**Table 8. Schedule of Study Procedures (Placebo Group)**

| **Visit** | **Visit 0** | **Visit 1** | **Visit 2** | **Visit 3** | **Unplanned Visits***** | ******** | **Visit 0** | **Visit 1** | **Visit 2** | **Visit 3** | **Unscheduled Visits** |
| --- | --- | --- | --- | --- | --- | --- | --- | --- | --- | --- | --- |
|  | **Screening** | **Day 0** | **Day 14** | **Day 28** | **Weekly phone visits** |  | **Screening** | **Day 0** | **Day 14** | **Day 28** | **Weekly phone visits** |
| Preliminary information | **X** |  |  |  |  |  | **X** |  |  |  |  |
| Informed consent | **X** |  |  |  |  |  | **X** |  |  |  |  |
| Screening for inc/excl. criteria | **X** |  |  |  |  |  | **X** |  |  |  |  |
| Physical Examination |  | **X** | **X** |  |  |  |  | **X** | **X** |  |  |
| Pregnancy Test |  | **X** | **X** |  |  |  |  | **X** | **X** |  |  |
| Total antikor (IgM+ IgG) testi | **X** |  |  |  |  |  | **X** |  |  |  |  |
| RT-PCR***** | **X** |  |  |  |  |  | **X** |  |  |  |  |
| Randomization |  | **X** |  |  |  |  |  | **X** |  |  |  |
| Vaccine administration |  | **X** | **X** |  |  |  |  | **X** | **X** |  |  |
| Collecting diary cards for safety observation and COVID-19 Symptoms |  |  | **X** | **X** |  |  |  |  | **X** | **X** |  |
| **COVID-19 Symptoms Follow-up by telephone visits |  |  |  |  | **X** |  |  |  |  |  | **X** |
| AdverseEvent and SAE Follow-up |  | **X** | **X** | **X** | **X** |  |  | **X** | **X** | **X** | **X** |
| Recording of concomitant medication |  | **X** | **X** | **X** | **X** |  |  | **X** | **X** | **X** | **X** |

* RT-PCR will be performed if any symptoms are developed during follow-up period

**Contacts will be made in a week after each vaccination and thereafter every week for the first 13 weeks after vaccination and every two weeks until 12 month-follow-up period completed

***Visits will be performed if any symptoms are developed

****After demonstrating clinical efficacy in the vaccine arm with interim analysis, the study vaccine will be administered to the subjects included in the placebo arm, and follow-up processes will continue as in the vaccine arm.

# SurveIllance for Adverse events

As mentioned earlier all participants will be observed for 30 minutes following vaccination and they will be advised to record daily any untoward medical events in the diary cards for 12 month after each dose and our staff will follow up daily over phone during this period. As well, IVRS system will call all participants by phone weekly to remind for informing any adverse event arises to investigation team in their sites or call center. A referral system will be established and all serious adverse events (SAE) will be managed and necessary hospitalization/treatment will be ensured according to the local regulations. All SAEs following vaccination will be reported to ethics committee and Ministry of Health and study sponsor within 24 hours of awareness. Safety follow up will be continued for 12 months after vaccination.

**Surveillance for seroresponses**

Blood samples will be taken from 1000 participants on Day 0, Day 14, Day 28, Day 42 and Day 120. COVID-19 specific neutralizing antibodies will be determined. We will determine the seroconversion rate, seropositive rate, GMT at different time points using standard methods.

**Surveillance for COVID disease**

Nasopharyngeal swabs will be collected from all participants in the visit mentioned in Study Method section. As well, participants who become symptomatic during follow-up will be instructed to call 24/7 Call centre or the study team member who will then advise on how to proceed with clinical testing for COVID-19 if necessary, as per the trial working instructions. All participants will be provided individually with a mobile set to contact call center/ study staffs. Participants will be instructed to call in any time for any kind of illness in designated phone numbers of the call Centre. Further, all the participants will be asked to inform study team members if they have any symptoms suggesting COVID-19 disease (fever, cough, shortness of breath, difficulty breathing, chill, nasal congestion, muscle pain, headache, sore throat, fatigue, vomiting, diarrhea, loss of smell (anosmia), loss of taste (ageusia). Participants will get weekly reminders by phone calls (IVRS calls and also text messages) to get in touch with the study team if they present with a fever or cough or shortness of breath and if they are admitted to hospital for any reason. At the COVID-19 testing visit, a nasal swab will be taken. Necessary management will be provided to all participants at the respective Covid-19 hospital. After symptom development, if first RT-PCR result is negative, second RT-PCR test will be repeated after 24 to 48 hours from the first RT-PCR test. If the second test is negative, subject will be followed up according to COVID-19 algorithm published by Ministry of Health. Symptomatic participants will be regularly reviewed over the phone and home visit and will be managed until the symptoms recover for patients not requiring hospitalization. A detailed CRF will be completed describing the clinical course and outcome for all hospitalized and non-hospitalized COVID-19 patients.

**24/7 Call Centre**

A 24/7 healthcare communication services will be established. Participants will be instructed to call in any time for any kind of illness in designated phone numbers of the call Centre. Proper management and referral system will be fixed through the call centre. When a participant call the call center the related site data coordinator will have a text message and site data coordinator will call back the participant and will understand the status of participant and schedule an appointment for a participant (if needed), arrange to visit participant home (if needed), arrange referral or any study related important information as required.

**Covid-19 Vaccine Trial Laboratory Protocol**

**Specimen Collection and Transportation**

Nasopharyngeal (NP) specimens will be collected from all cases. NP flocked swab will be collected and placed into viral transport medium (VTM). The VTM with swab stick will be placed in ziploc bags will be carried in a cooler box with ice pack (maintaining 2 to 8˚C) and transported in cooler box to determined central laboratories (Figure 1 and 2).


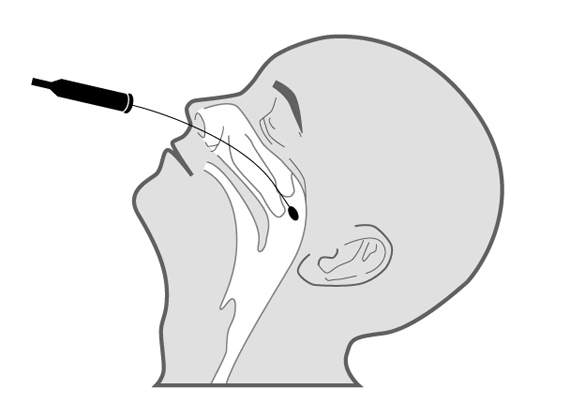


**Figure -2 Specimen collection**


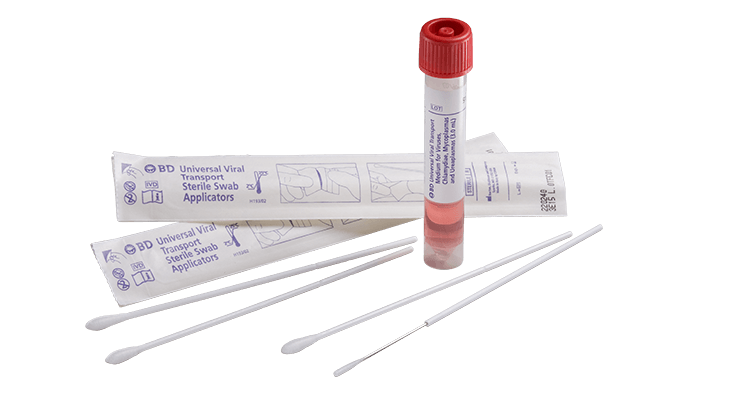


**Figure - 3: Viral Transport Media**

**Diagnosis of SARS-CoV-2**

**Sample processing**

NP samples will be processed in a dedicate BSL2 laboratory for BSL3 practices, under a certified Class II biological safety cabinet (BSC). Once a clinical sample has been treated with lysis buffer for RNA extraction, the samples can be moved to a less restrictive environment to complete the RNA extraction and real time RT-PCR.

**RNA Extraction**

An aliquot of 140μl of specimen will be added to 560μl of lysis buffer (Qiagen Viral Mini kit). RNA will be extracted as described by the manufacturer’s protocol and immediately processed for RT-PCR. The remaining nucleic acid will be stored at -80° C for sequencing purposes.

**Real-time RT-PCR (rRT-PCR)**

Real-time reverse transcriptase-PCR (rRT-PCR) detection is currently gold standard for the detection of coronavirus because of its advantages as a specific, and simple quantitative assay. Moreover, rRT-PCR is more sensitive for the diagnosis in early infection. The primers and probes which can detect at least two genes of SARS-CoV-2 will be used. The technique utilizes a one step protocol with specific primers designed to amplify a portion of the genome that contains a target PCR sequence. Non-extendible fluorogenic hydrolysis/Taqman probes monitor the target PCR product formation at each cycle during the PCR reaction.

Briefly, reaction mix will be prepared by pipetting: H_2_O, kit-supplied reaction buffer, dNTPs, 25mM MgCl_2_, and primers in a nuclease free microcentrifuge tube. Next, the RNase inhibitor and enzyme will be added and the probe at last. Template RNA will be added to the reaction mix and inserted into thermal cycler. The reaction will be carried out using a thermal cycler. The system uses an internal passive reference molecule which acts as a normalization factor for fluorescent emission detected in the samples. Results will be analyzed by the software provided by the manufacturer of the thermal cycler. Comparative viral load will be calculated using ct values of consecutive specimens.

Viral load will be determined in a passive method from CT values of rRT-PCR.

Blood will also be processed in BSL2 facilities in certified biosafety cabinet. Serum/plasma separated from blood will be stored at -80^o^C for immunological analysis.

**SARS-CoV2-Neutralizing Antibody Determination:**

Virus Neutralization Test technique will be applied for determination of SARS-CoV2-Neutralizing Antibody in Ankara University Virology Department BSL -3 Laboratory. After inactivating blood serum samples at 56 °C for 30 minutes, they will be mixed with 100DKID50 SARS-CoV2 (approximately 1:100 diluted virus) at the same volume and left at 37 °C for one hour for neutralization. At the end of this duration Vero E6 cells will be inoculated with this mixture and incubated at 37°C for 96 hours. Test will be checked for virus and cell control in cell culture microscope. Test will be evaluated when 100% of SARS-CoV2 specific cpe is observed in virus control section.

**Cellular Immunization Test Method**

After vaccination, 30 mL peripheral blood will be collected from individuals. Peripheral blood mononuclear cells will be obtained by density separation from collected blood. Then, CD14+ monocytes will be purified by MACS (magnetic-activated cell sorting) and the remaining cells will be frozen and stored in liquid nitrogen. Purified monocytes will be incubated with IL-4 and GM-CSF for five days. On the fifth day, the vaccine preparation, the recombinant SARS-CoV-2 spike glycoprotein and the tetanus toxin for positive control will be added in separate conditions and incubated for two days in order to perform antigen loading of dendritic cells. At the end of the seventh day, validation will be performed in flow cytometry with monoclonal antibodies, anti-CD14, anti-CD1a, anti-CD83 and anti-CD11c, to confirm dendritic cell conversions. After antigen loading to dendritic cells, frozen peripheral blood mononuclear cells of the same individual are thawed, monoclonal antibodies, anti-CD19, anti-CD56, anti-CD45RA and anti-CD45RO, and memory (CD19-CD56-CD45RO+) and naive T cells (CD19-CD56-CD45RA+) will be purified by FACS (fluorescence-activated cell sorting) method. Purified T cells will be labeled with CFSE proliferation dye and the first signal will be given with anti-CD3 monoclonal antibody. After this, T cells will be co-cultured with the vaccine preparation, recombinant SARS-CoV-2 spike glycoprotein, and dendritic cells loaded with tetanus toxin for 72 hours in combinations. At the end of the incubation period, activation levels of T cells will be determined in flow cytometry by marking with monoclonal antibodies, anti-CD4, anti-CD25, anti-CD107a, anti-4-1BB and anti-CD62L. Also, proliferation percentages of T cells will be determined according to dilution of CFSE proliferation dye in flow cytometry. Supernatant will be collected from co-cultures and measured by ELISA in order to measure IFN-g and IL-2 levels in co-cultures.

# Safety Assessment

Safety will be assessed by the frequency, incidence and nature of AEs and SAEs reported during the study.

**Definitions**

**Adverse Event (AE)**

An AE is any untoward medical occurrence in a volunteer, which may occur during or after administration of an investigation product (IP) and does not necessarily have a causal relationship with the intervention. An AE can therefore be any unfavorable and unintended sign (including any clinically significant abnormal laboratory finding or change from baseline), symptom or disease temporally associated with the study intervention, whether or not considered related to the study intervention.

**Serious Adverse Event (SAE)**

An SAE is an AE that results in any of the following outcomes, whether or not considered related to the study intervention.

- Death
- Life-threatening event (i.e., the volunteer was, in the view of the Investigator, at immediate risk of death from the event that occurred).
- Persistent or significant disability or incapacity (i.e., substantial disruption of one’s ability to carry out normal life functions).
- Hospitalization or prolongation of existing hospitalization, regardless of length of stay, even if it is a precautionary measure for continued observation. Hospitalization (including inpatient or outpatient hospitalization for an elective procedure) for a pre-existing condition that has not worsened unexpectedly does not constitute a serious AE.
- An important medical event (that may not cause death, be life threatening, or require hospitalization) that may, based upon appropriate medical judgment, jeopardise the volunteer and/or require medical or surgical intervention to prevent one of the outcomes listed above. Examples of such medical events include allergic reaction requiring intensive treatment in an emergency room or clinic, blood dyscrasias, or convulsions that do not result in inpatient hospitalization.
- Congenital anomaly or birth defect.

**Serious Adverse Reaction (SAR)**

An AE that is both serious and, in the opinion of the reporting Investigator or Sponsors, believed to be possibly, probably or definitely due to an IP or any other study treatments, based on the information provided.

**Causality**

For every AE, an assessment of the relationship of the event to the administration of the vaccine will be undertaken by investigator team. An interpretation of the causal relationship of the intervention to the AE in question will be made, based on the type of event; the relationship of the event to the time of vaccine administration; and the known biology of the vaccine therapy. Alternative causes of the AE, such as the natural history of pre-existing medical conditions, concomitant therapy, other risk factors and the temporal relationship of the event to vaccination will be considered and investigated. Causality assessment will take place during planned safety reviews, and at the final safety analysis, except for SAEs, which should be assigned by the reporting investigator, immediately with 24 hours of awareness of the event.

**Table 9. Guidelines for assessing the relationship of vaccine administration to an AE.**

| 0 | **No Relationship** | No temporal relationship to study product ***and***  Alternate aetiology (clinical state, environmental or other interventions); ***and***  Does not follow known pattern of response to study product |
| --- | --- | --- |
| 1 | **Unlikely** | Unlikely temporal relationship to study product ***and***  Alternate aetiology likely (clinical state, environmental or other interventions) ***and***  Does not follow known typical or plausible pattern of response to study product. |
| 2 | **Possible** | Reasonable temporal relationship to study product; ***or***  Event not readily produced by clinical state, environmental or other interventions; ***or***  Similar pattern of response to that seen with other vaccines |
| 3 | **Probable** | Reasonable temporal relationship to study product; ***and***  Event not readily produced by clinical state, environment, or other interventions ***or***  Known pattern of response seen with other vaccines |
| 4 | **Definite** | Reasonable temporal relationship to study product; ***and***  Event not readily produced by clinical state, environment, or other interventions; ***and***  Known pattern of response seen with other vaccines |

**Reporting Procedures for All Adverse Events**

All local and systemic AEs following each vaccination will observed by the Investigator or reported by the participants, whether or not attributed to study vaccine, will be recorded in CRF, diaries and electronic study database throughout the study period. All AEs that result in a participant’s withdrawal from the study will be followed up until a satisfactory resolution occurs. SAEs will be collected throughout the entire study period.

**Assessment of severity**

The severity of clinical adverse events will be assessed according to scales based on FDA toxicity grading scales for vaccine clinical trials, as shown in the tables below.

**Table 10. Severity grading criteria for local adverse events**

|  | **Grade 1** | **Grade 2** | **Grade 3** | **Grade 4** |
| --- | --- | --- | --- | --- |
| Pain | Not affecting or slightly affecting physical activity | Affecting physical activity | Affecting daily life | Loss of basic self-care ability, or hospitalization |
| Induration*# | Diameter 2.5 to <5 cm or area 6.25 to <25 cm^2^ without affecting or slightly affecting daily life | 5 to <10 cm in diameter or 25 to <100 cm^2^ in area or affecting daily life | Diameter ≥10 cm or area ≥100 cm^2^ or ulceration or secondary infection or phlebitis or aseptic abscess or wound drainage or seriously affecting daily life | Abscess, exfoliative dermatitis, dermal or deep tissue necrosis |
| Swelling # | Diameter 2.5 to <5 cm or area 6.25 to <25 cm^2^ without affecting or slightly affecting daily life | 5 to <10 cm in diameter or 25 to <100 cm^2^ in area or affecting daily life | Diameter ≥10 cm or area ≥100 cm^2^ or ulceration or secondary infection or phlebitis or aseptic abscess or wound drainage or seriously affecting daily life | Abscess, exfoliative dermatitis, dermal or deep tissue necrosis |
| Redness# | Diameter 2.5 to <5 cm or area 6.25 to <25 cm^2^ without affecting or slightly affecting daily life | 5 to <10 cm in diameter or 25 to <100 cm^2^ in area or affecting daily life | Diameter ≥10 cm or area ≥100 cm^2^ or ulceration or secondary infection or phlebitis or aseptic abscess or wound drainage or seriously affecting daily life | Abscess, exfoliative dermatitis, dermal or deep tissue necrosis |
| Rash* # | Diameter 2.5 to <5 cm or area 6.25 to <25 cm^2^ without affecting or slightly affecting daily life | 5 to <10 cm in diameter or 25 to <100 cm^2^ in area or affecting daily life | Diameter ≥10 cm or area ≥100 cm2 or ulceration or secondary infection or phlebitis or aseptic abscess or wound drainage or seriously affecting daily life | Abscess, exfoliative dermatitis, dermal or deep tissue necrosis |
| Pruritus | Itching at injection site, relieved within 48 hours | Itching at injection site, did not alleviate within 48 h after treatment | Affecting daily life | NA |

* In addition to directly measuring the diameter for grading evaluation, sclerosis and rash should also record the progress of measurement, results.

# The maximum measured diameter or area should be used for induration and swelling, rash and red; evaluation and grading should be based on functional grade and actual measurement results, and higher grading indicators should be selected.

**Table 11. Severity grading criteria for systemic adverse events and vital signs**

|  | **Grade 1** | **Grade 2** | **Grade 3** | **Grade 4** |
| --- | --- | --- | --- | --- |
| Diarrhea | Mild or transient, 3-4 times/day, abnormal stool, or mild diarrhea lasting less than 1 week | Moderate or persistent, 5-7 times/day, abnormal stool, or diarrhea >1 week | >7 times/day, abnormal stool, or hemorrhagic diarrhea, orthostatic hypotension, electrolyte imbalance, requiring intravenous infusion >2L | Hypotensive shock, hospitalization |
| Decreased Appetite | Decreased appetite, not affecting food intake | Decreased appetite, reduced food intake, not affecting body weight | Decreased appetite, and significantly reduced body weight | Need intervention (such as gastric tube feeding, parenteral nutrition) |
| Vomiting | 1-2 times/24 hours without affecting activity | 3-5 times/24 hours or affecting activity | >6 times within 24 hours or requiring intravenous fluid infusion | Hospitalization or other nutrition routes due to hypotensive shock |
| Nausea | Transient (<24 hours) or intermittent and basically normal food intake | Persistent nausea leads to reduced food intake (24-48 hours) | Persistent nausea leads to almost no food intake (>48 hours) or requires intravenous fluids | Life threatening (e.g., hypotensive shock) |
| Muscle pain (non-inoculated site) | Does not affect daily activities | Slightly affects daily activities | Severe muscle pain, seriously affects daily activities | Emergency or hospitalization |
| Joint Pain | Mild pain, not affecting daily activities | Moderate pain, requiring analgesics and/or pain interferes with functioning, yet not affecting daily activities | Severe pain, seriously affecting daily activities | Emergency or hospitalization |
| Headache | Not affecting daily activities, no treatment required | Transient, slightly affecting daily activities, may need treatment or intervention | Seriously affecting daily activities, need treatment or intervention | Intractability, need emergency or hospitalization |
| Cough | Transient, no treatment required | Persistent cough, effective treatment | Paroxysmal cough, uncontrolled treatment | Emergency or hospitalization |
| Fatigue | Normal activity is weakened <48 hours, without affecting the activity | Normal activity is weakened by 20%~50%>48 hours, slightly affecting the activity | Normal activity is weakened by >50%, seriously affecting daily activities, unable to work | unable to take care of oneself, emergency or hospitalization |
| Pruritus | Mild or localized; topical intervention indicated | Widespread and intermittent; skin changes from scratching (e.g., edema, papulation, excoriations, lichenification, oozing/crusts); oral intervention indicated; limiting instrumental activities of daily living | Widespread and constant; limiting self care activities of daily living or sleep; systemic corticosteroid or immunosuppressive therapy indicated | ----- |
| Skin rash (exanthema)† | Present, but asymptomatic | Symptomatic (pruritus/pain), but interferes only slightly with daily activities | Symptomatic, prevents daily activities | Emergency or hospitalization |
| Allergic reaction | Systemic intervention not indicated | Oral intervention indicated | Bronchospasm; hospitalization indicated for clinical sequelae; intravenous intervention indicated | Life-threatening consequences; urgent intervention indicated |
| **Vital Signs** |  |  |  |  |
| Fever (oral temperature) | 37.5～＜38.2℃ | 38.2～＜38.7℃ | ≥38.7℃ | ≥39.7℃, Lasting more than 3 days |

†Specify if the skin rash is located in any specific body part or if it is widespread.

The severity of the unsolicited clinical adverse events will be classified through a numeric scale of 1 to 5 , which was created based on the “Guidelines for grading scale of adverse events in vaccine clinical trials, 2019” of the National Medical Products Administration, China.

**Table 12. Severity grading criteria for for unsolicited adverse events*.***

| ***GRADE 1 (Mild)*** | *Transient (< 48 hours) or mild discomfort; no medical intervention/therapy required* |
| --- | --- |
| ***GRADE 2 (Moderate)*** | *Mild to moderate limitation in activity - some assistance may be needed; no or minimal medical intervention/therapy required* |
| ***GRADE 3 (Severe)*** | *Marked limitation in activity, some assistance usually required; medical intervention/therapy required, hospitalizations possible* |
| ***GRADE 4 (Life-threatening)*** | *Extreme limitation in activity, significant assistance required; significant medical intervention/therapy required, hospitalization or hospice care probable* |
| ***GRADE 5*** | *Death* |

**Reporting Procedures for SAEs**

Any SAE must be reported to Data and Safety Monitoring Board (DSMB), Ethics committee, MoH, sponsor and clinical study monitor within 24 hours of the investigator’s first knowledge of the event, regardless of the presumed relationship to the investigational product. The investigator or qualified designee must complete the SAE Report Form, sign, and transmit the completed form to DSMB, study sponsor and clinical study monitor.

Initial reports of SAEs may be reported via fax or e-mail. Initial reports via telephone **must** be supported by transmission of documentation (paper or electronic) signed (physically or electronically) by the investigator or a qualified sub-investigator **within 24 hours** of notification. When additional follow-up information becomes available, a follow-up SAE report (depending on the qualified event) must be completed, signed by the investigator or a qualified sub-investigator and transmitted as soon as possible. The investigator is responsible for obtaining detailed information to support all SAE reports, including records of inpatient and outpatient care, laboratory reports, and autopsy or medical examiner reports.

# Data Safety MonItorIng Plan (DSMP)

**Data and Safety Monitoring Board**

The study will be monitored by an Independent Data and Safety Monitoring Board (DSMB). The committee is responsible for the monitoring and review of the safety and conduct of the trial. The DSMB is expected to convene meeting at the start of the study, near the middle of the study and at study completion. In addition, the committee will convene at any other time point during the study if found necessary to share / get advice of the DSMB to ensure participants’ safety.

**Data and Safety Monitoring Board Plan**

Academicians from different institutions were selected for the team to work in the independent data monitoring committee. In this context, specialists experienced in infectious diseases and clinical microbiology, public health-epidemiology, social pediatric, vaccination/adverse effects will be included in the group. This committee will monitor the quality of evidence, adverse event following, revisions in line with the current literature, individual privacy, and data reliability from the planning stage to the end of the study. It will follow that the practices are carried out in accordance with Good Clinical Practice and Human Rights Declaration conditions. In the study to be blinded, if there is an improvement for or against any of the arms, the committee will be able to make an opinion about stopping the vaccination or giving the placebo group the chance to be followed by vaccinating based on personal benefit. Similarly, according to the evaluation of the results of the K1 branch, which will first start in health care workers and risked population, the committee will make an evidence-based assessment and will give an opinion on whether the K2 study should be done, if necessary revised or not. When necessary, the Coordinator may request data confirmation/additional data by directly contacting the CRO and/or centers.

**A communication network only for members (WhatsApp group and closed email group)** will be established between the Independent Data Monitoring Committee. It is essential to use this communication network to strengthen daily or emergency communication.

**A web page** will be prepared to be accessed by Independent Data Monitoring Committee, Administrative Responsible, Coordinator and CRO members and a page will be created in which new safety data that may arise in the entire adverse reaction, pandemic process or study and new data on the effect are updated daily. This page can be followed by the members regularly and when requested.

Follow-up involving health care workers (K1) and risked population is particularly valuable. Safety monitoring (as it occurs and after it is confirmed) in a cohort follow-up, which is the first period of vaccination administration, should be updated on the website. On a weekly basis, the CRO will present this data in a table form by groups and impact groups. Intermediate efficacy and safety assessments (without disrupting blinding) should be reported cumulatively on a monthly basis. The Independent Data Monitoring Committee will discuss this data through weekly online sessions; if it deems necessary, the Committee will be able to request a meeting with the Coordinator; the meeting time may be extended depending on the increase in the number of subjects included in the study and the reliability results; it can be calculated as expected one-hour sessions. Independent Data Monitoring Committee may request new members (from fields such as ethics, law, communication, etc.) to be added to the team, depending on the results of reliability and effectiveness. It is appropriate to justify this request and accept it by majority of votes.

According to the protocol, participants were defined as “2 separate cohorts”. The first cohort is the cohort of Health care workers and 1300 people will be included in this cohort. After evaluation of interim safety report which will be prepared after 500 subjects are randomized in K-1 cohort, K-2 will be initiated to be included in the study. Cohort 2 consists of the population at normal risk and it is planned to include 11,150 people in this group.

Following the collection of K1 study group data or when a total of 40 COVID-19 cases occur in both arms, **whichever occurs first**, the effect (RR, RD, NNT and NNT values) will be calculated by evaluating the research data, and the adverse effect number, type and grade distribution will be made. If the Independent Data Monitoring Committee examines this assessment and it is concluded that the adverse effects are within acceptable limits, the second cohort will be initiated. Otherwise, the sample size of the second cohort can be changed, and the inclusion and exclusion criteria can be changed and additional test or examinations can be requested. If the protection percentage is found to be above 60% in the study interim analysis and the adverse effect is below the determined rate (it will be determined separately for Grade 1-2 and Grade 3-4), this situation will be discussed with Data Monitoring Committee and the investigators who are determined by Committee and will be finalized according to the decisions to be made. In both cases, progress will be achieved by sharing the results and justifications with the ethics committee and MoH. All individuals participating in the study will be followed up for adverse effects for at least 360 days after vaccine/placebo administration/transition from placebo to vaccine. If information is produced regarding the development of long-term complications/sequelae of COVID-19 or vaccine in the process, a follow-up recommendation for over 1 year can be made with a second protocol. This situation can be examined with a new study.

**Monitoring**

In addition to DSMB, TÜSEB as the sponsor of the study, is responsible for ensuring the proper conduct of the study, in accordance with the Declaration of Helsinki (Amended Fortaleza, Brazil, 2013) and Good Clinical Practices (GCP) including, but not limited to, protocol adherence and the validity of the data recorded in the database. For the purposes of this study, TÜSEB may transfer responsibility for the clinical monitoring to Independent clinical monitors of contract research organization (CRO) who may monitor on-site or remotely. Independent clinical monitors are responsible for ensuring that the site(s) prepare complete, accurate, legible and well-organized clinical study data. On-site monitoring inspections will be routinely performed in order to review data entry of source documentation directly captured on paper and transcribed into the system, to ensure protocol adherence, to assess site operational capabilities and to perform other monitoring activities that cannot be performed remotely. In addition, clinical monitors will provide ongoing support to ensure the investigator’s continued understanding of all applicable regulations concerning the clinical evaluation of the investigational vaccine, and the proper execution of the protocol, as well as the investigator’s reporting responsibilities.

The clinical study sites will be monitored periodically for database accuracy and completeness, adherence to the protocol, regulatory compliance, safety reporting, clinical trial material accountability, and the maintenance of comprehensive source documents. Where applicable, the database will be checked against applicable source documents to verify completeness and accuracy. When data entry has been completed by the appropriate study staff, source document verified and monitored by CRO representatives, and reviewed by the investigator, the investigator should sign and date the *Investigator Signature Page*.

**Data Management and security measure**

eCRFs (Electronic Case Report Forms/Electronic Data Capture Forms) will be developed for data entry in study sites. Necessary data management and reporting tools will be also developed for administration and reporting the project status.

The complete set of eCRFs will be online. Medical officers (MOs) / Nurses/ Data Management officers (as per project defined role) will use eCRF system online and they directly enter the data using the eCRFs. These data will be stored in the server online. All these data will be checked and verified by the senior physician (SP)/ MO. After checking and verifying the eCRFs by SP/MO, they sign in eCRFs and make complete the forms and save the record as complete.

The study site will maintain appropriate medical and research records for this trial, in compliance with ICH-GCP, regulatory, sponsor and institutional requirements for the protection of confidentiality of participants. The site will permit authorized representatives of the sponsor(s), ERC/DSMB monitor, auditor and regulatory agencies to examine (and when required by applicable law, to copy) clinical records for the purposes of quality assurance reviews, audits, and evaluation of the study safety progress. Based on the user roles, user can access the particular part of the data or whole database eCRF System. In the project initiation meeting, persons and their roles and responsibilities will be defined. Based on the roles and responsibilities, user will be created and roles will be assigned to the respective persons.

Electronic Data Capture (EDC) is a web-based application to capture data for clinical research and create databases and projects. It is highly secure and intuitive to use. This server is 24hrs online, fault tolerant  and automatic back-up system. There are logging system for collecting every events/action logs  taken place in the system. At the user end, we have daily, weekly and monthly back-up system to back-up all the data in a local computer. The data will be stored in a main server found in Turkey.

Data can be downloaded from the server in SAS/ SPSS/ STATA/ CSV format as per project need.

**Source documents**

Source documents include but are not limited to:

- Documentation of the study eligibility evaluation.
- Signed informed consent documents.
- Visit documentation that includes dates of study visits and dates of study vaccinations.
- Reported laboratory results.
- AE evaluations.
- Concomitant medications.
- Diary Card
- Certified copies of hospital records.

**Data Analysis**

All analyses will be performed only on cleaned, locked, and frozen data sets. The data will be analysed by statisticians. Statistical packages that may be used for the analysis include SPSS and STATA. Case Report Forms (CRFs) will be developed, pretested, and finalized for acquisition of relevant data. Incidence of COVID-19 will be calculated as total number of episodes (lab-documented or clinical illnesses)/person-years for at-risk population * 1000. Protective efficacy of the vaccine will be calculated as [1- (rate ratio of COVID-19 infection in vaccines vs. placebo recipients) X 100]. This will be done according to per-protocol (PP) analysis. Risk factors (e.g age, sex, pre-existing diseases, duration and nature of job, living conditions, comorbidity.) for development of the disease and vaccine failure will be determined by comparing between cases and controls (without disease) using appropriate tests. Continuous variables will be compared between groups by Mann-Whitney U test and within groups by the paired T-test, and associations of continuous variables will be assessed by Pearson's correlation coefficient or the Spearman rank correlation test. The chi square or Fisher's exact test will be used to compare proportions between groups. Categorical data on covariates (explanatory variables) associated with specific outcomes may also be analysed as Chi-Square tables (e.g. Kruskal-Wallis Chi squared test of equality of populations) or as 2 x 2 and 2 x X tables in univariate and multivariate analysis, with calculation of odds ratios with 95% confidence intervals, and modelled using unconditional logistic regression, using the outcome of interest as the outcome variable and all other covariates, such as demographic, patient history, clinical data, as explanatory variables.

An interim analysis will be planned after 40 confirmed COVID-19 cases.

# EthIcal Assurance for ProtectIon of Human rIghts

**Statement of Compliance**

The study described in this protocol will be conducted in compliance with the protocol. Ethical Committee of Hacettepe University will review and approve the protocol prior to study start. Documentation of the approval by these bodies will be kept in the PI’s study file.

**Potential Risks and Benefits**

Effects of venous blood draws: Risks of bruising, bleeding, infection and fainting can accompany these procedures. Study-related venous blood collection for safety assessment and research purpose will be obtained following standard blood collection technique.

Potential risks to study personnel: The main risks to study personnel are from accidental exposure to droplets, and body fluids of patients. SOPs for staff safety are used in clinical and laboratory areas using Personal Protective Equipments (PPE) including sharps management, hazardous waste management, etc. Universal precautions are used for handling all body fluids.

Known Potential Benefits: Participants may not receive any direct benefit from their participation in the study. However, if any harm occurs due to participation, participants will be referred for prompt evaluation and treatment by the appropriate study medical officer/hospital doctor. The information gained from the proposed work is beneficial for the study participants in the future, others living in the community, as well as public health professionals and policymakers in Bangladesh.

**Participant confidentiality**

Participant confidentiality is held strictly in trust by the participating investigators and their staff. This confidentiality is extended to cover testing of biological samples, in addition to the demographic information relating to participating participants. The study protocol, documentation, data and all other information generated will be held in strict confidence. Study records including case record forms and consent forms will be stored in locked cabinets in secure facilities, only accessible to authorized investigators, and/or in password-protected devices and computers.

All participants will be assigned a unique study identification (ID) number. This number will be used to label all biologic samples and study-related documents except informed consent/assent forms which will contain the participant’s identifier and will be stored in secured space as described below. All data generated from each study sample will be linked to the coded, unique ID and associated study data.

Study data will be stored on password protected computers at the study site and later in main server of Omega CRO, with restricted access to specified study personnel. Data generated from study participants may be submitted to publicly available databases, but will not be linked to any identifying information from study participants. Participants will not be identified in any publications resulting from the study.

The data will be used by the PI and study collaborators to assess safety and efficacy of the study drugs.

**Future Use of Stored Specimens**

Blood/nasal samples can be used in future studies. Specimens will be labelled with a coded, unique identifier and will not contain identifying information.

**Informed Consent Process**

The principles of informed consent in the current edition of the Declaration of Helsinki will be implemented before any protocol-specified procedures are carried out.

Information about the study will be given to prospective participants in both oral and written forms whenever possible. The written consent documents will embody the elements of informed consent: purpose of study, background, methods, why the participant is invited, risk and benefits, voluntariness of participation, compensation, privacy, confidentiality, anonymity, contact address of investigators, ethical review committee etc. The consent form will be read out to the participants, invite them to ask any question regarding the study, reply to their queries with their full satisfaction. Independent witnesses will be used to attest that potential participants have understood the contents of the informed consent document.

We consider informed consent to be a dynamic, ongoing process, with continuous availability of investigators to answer any questions that arise in the course of the study and to ensure that participants understand study procedures. Contact information is provided in terms of local physicians who can be visited directly and who can themselves reach the investigators directly or by telephone or email.
